# Maternal PlGF and umbilical Dopplers predict pregnancy outcomes at diagnosis of early-onset fetal growth restriction

**DOI:** 10.1172/JCI169199

**Published:** 2023-09-15

**Authors:** Rebecca Spencer, Kasia Maksym, Kurt Hecher, Karel Maršál, Francesc Figueras, Gareth Ambler, Harry Whitwell, Nuno Rocha Nené, Neil J. Sebire, Stefan R. Hansson, Anke Diemert, Jana Brodszki, Eduard Gratacós, Yuval Ginsberg, Tal Weissbach, Donald M. Peebles, Ian Zachary, Neil Marlow, Angela Huertas-Ceballos, Anna L. David

**Affiliations:** 1 UCL Elizabeth Garrett Anderson Institute for Women’s Health, University College London, London, United Kingdom.; 2 Leeds Institute of Cardiovascular and Metabolic Medicine, University of Leeds, Leeds, United Kingdom.; 3 Department of Obstetrics and Fetal Medicine, University Medical Center Hamburg-Eppendorf, Hamburg, Germany.; 4 Department of Obstetrics and Gynaecology, Institute of Clinical Sciences Lund, Skane University Hospital, Lund University, Lund, Sweden.; 5 Institut D’Investigacions Biomèdiques August Pi í Sunyer, University of Barcelona, Barcelona Center for Maternal-Fetal and Neonatal Medicine, Barcelona, Spain.; 6 Department of Statistical Science, University College London, London, United Kingdom.; 7 National Phenome Centre and Imperial Clinical Phenotyping Centre, Department of Metabolism, Digestion and Reproduction and; 8 Section of Bioanalytical Chemistry, Division of Systems Medicine, Department of Metabolism, Digestion and Reproduction, Imperial College London, London, United Kingdom.; 9 Population, Policy and Practice Department, Great Ormond Street Institute of Child Health, University College London, London, United Kingdom.; 10 Department of Obstetrics and Gynecology, Rambam Medical Centre, Haifa, Israel.; 11 Department of Obstetrics and Gynecology, Sheba Medical Center Tel Hashomer, Tel Aviv, Israel.; 12 Division of Medicine, Faculty of Medical Sciences, University College London, United Kingdom.; 13 Neonatal Department, University College London Hospitals NHS Foundation Trust, London, United Kingdom.

**Keywords:** Reproductive Biology, Clinical practice, Diagnostics, Obstetrics/gynecology

## Abstract

**BACKGROUND:**

Severe, early-onset fetal growth restriction (FGR) causes significant fetal and neonatal mortality and morbidity. Predicting the outcome of affected pregnancies at the time of diagnosis is difficult, thus preventing accurate patient counseling. We investigated the use of maternal serum protein and ultrasound measurements at diagnosis to predict fetal or neonatal death and 3 secondary outcomes: fetal death or delivery at or before 28+0 weeks, development of abnormal umbilical artery (UmA) Doppler velocimetry, and slow fetal growth.

**METHODS:**

Women with singleton pregnancies (*n* = 142, estimated fetal weights [EFWs] below the third centile, less than 600 g, 20+0 to 26+6 weeks of gestation, no known chromosomal, genetic, or major structural abnormalities) were recruited from 4 European centers. Maternal serum from the discovery set (*n* = 63) was analyzed for 7 proteins linked to angiogenesis, 90 additional proteins associated with cardiovascular disease, and 5 proteins identified through pooled liquid chromatography and tandem mass spectrometry. Patient and clinician stakeholder priorities were used to select models tested in the validation set (*n* = 60), with final models calculated from combined data.

**RESULTS:**

The most discriminative model for fetal or neonatal death included the EFW *z* score (Hadlock 3 formula/Marsal chart), gestational age, and UmA Doppler category (AUC, 0.91; 95% CI, 0.86–0.97) but was less well calibrated than the model containing only the EFW *z* score (Hadlock 3/Marsal). The most discriminative model for fetal death or delivery at or before 28+0 weeks included maternal serum placental growth factor (PlGF) concentration and UmA Doppler category (AUC, 0.89; 95% CI, 0.83–0.94).

**CONCLUSION:**

Ultrasound measurements and maternal serum PlGF concentration at diagnosis of severe, early-onset FGR predicted pregnancy outcomes of importance to patients and clinicians.

**TRIAL REGISTRATION:**

ClinicalTrials.gov NCT02097667.

**FUNDING:**

The European Union, Rosetrees Trust, Mitchell Charitable Trust.

## Graphical abstract

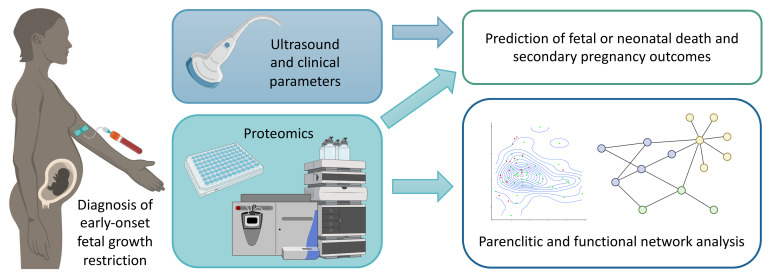

## Introduction

The survival and growth of a fetus depends on placental provision of nutrients and waste exchange with the mother. When this system is impaired by inadequate transformation of the uteroplacental circulation or deficits in the structure or function of the placenta, the fetus fails to reach their growth potential (1, 2). The resulting fetal growth restriction (FGR) may be diagnosed antenatally on the basis of an ultrasound-determined low estimated fetal weight (EFW) for gestational age — either below the third centile or below the tenth centile with abnormal Doppler ultrasound indices in the uterine artery (UtA) and/or umbilical artery (UmA) (3–5). Early-onset FGR, occurring before 32 weeks of gestation, carries significant risks of stillbirth, neonatal morbidity and mortality, neurodevelopmental impairment, and long-term health problems (6–13). There is currently no treatment that can improve fetal growth in utero; instead, management involves monitoring the pregnancy and timing delivery to balance the risks of stillbirth and prematurity (4, 14–16).

An important question when developing novel therapies for early-onset FGR is which pregnancies to include in early-phase clinical trials. There is a balance to be struck between identifying pregnancies that are sufficiently severely affected to justify the possible risks of the intervention but not so severely affected that there is no potential to determine efficacy. Numerous studies have investigated predictive markers for the development of FGR (17–22), but far fewer have studied the prediction of pregnancy outcome when early-onset FGR is diagnosed. The inability to predict pregnancy outcomes leaves pregnant patients and their partners with a considerable burden of uncertainty (23, 24). It also limits clinicians’ abilities to personalize management and counseling, including about termination of the pregnancy where this is a legal option.

The EVERREST project aims to carry out a phase I/IIa trial of maternal VEGF gene therapy for early-onset FGR (25). The greatest potential for benefit is in pregnancies at the threshold of viability, for which our current management option, preterm delivery, is not possible or is very high risk. In preparation for a clinical trial of a novel therapeutic, we established a multicenter prospective study to characterize the natural history of early-onset FGR, choosing an extreme phenotype in which the EFW was below the third centile and less than 600 g between 20+0 and 26+6 weeks of gestation (henceforth referred to as severe, early-onset FGR) (26). Neonatal outcomes for the live-born babies from this case series have recently been published (27).

The aim of this work was to prospectively identify and validate ultrasound and serum biochemical factors that could be used to predict fetal or neonatal death in pregnancies affected by severe, early-onset FGR. These could subsequently be used to select the most appropriate women for a first-in-human study of a novel therapeutic to treat FGR and to better counsel women and their partners about pregnancy outcome. To this end, we asked patients and clinicians to assess the value of our primary and secondary outcomes, on the basis of which we then selected models for validation. Unsupervised parenclitic network analysis by pregnancy outcome and functional network analysis of proteins associated with pregnancy outcome were also performed to maximize the utility of the proteomics data with the aim of providing insights into the underlying pathophysiology.

## Results

**Figure 1:**
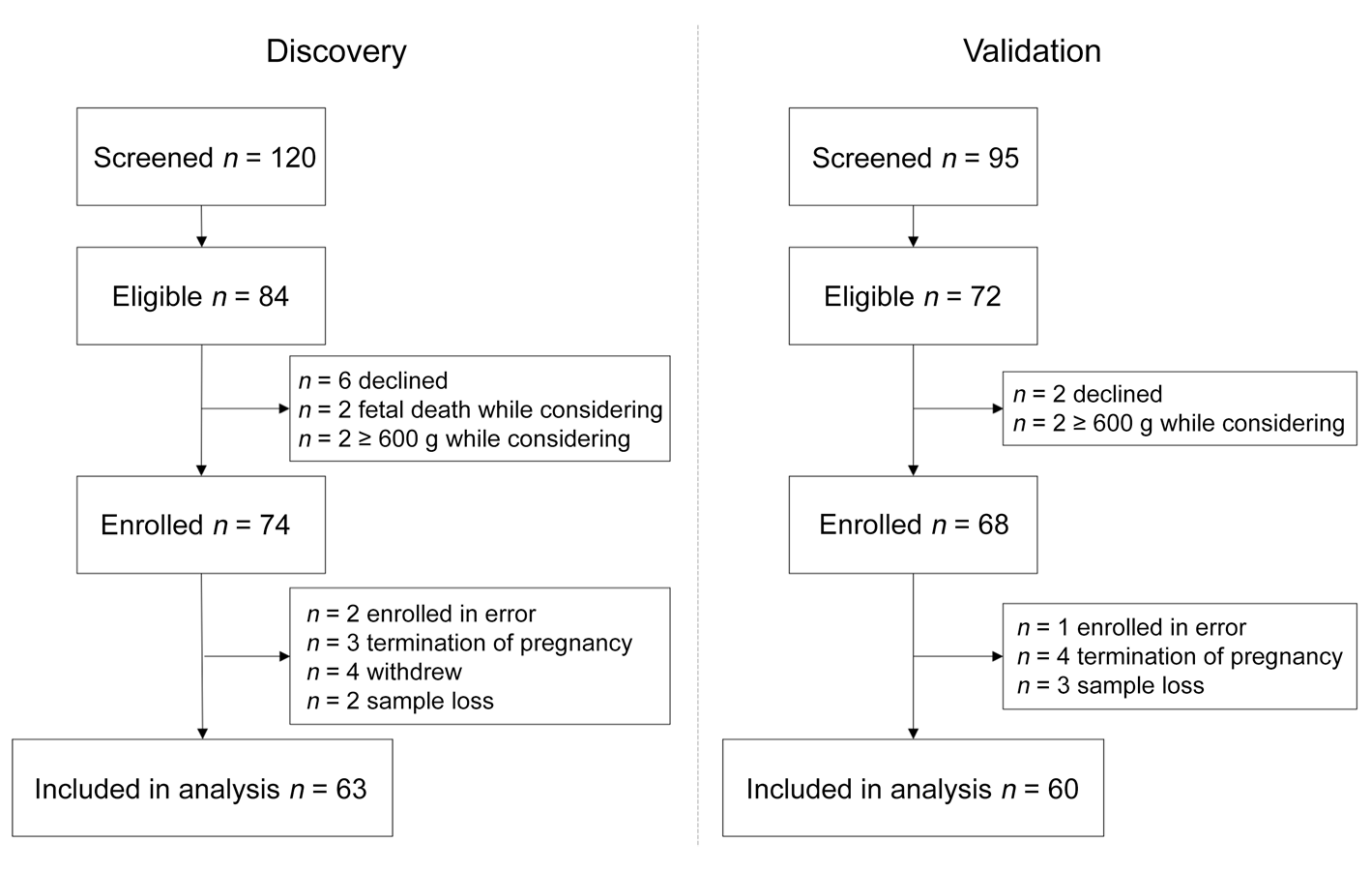
Flow diagram of participant eligibility and enrollment across the 4 EVERREST Prospective Study centers from March 10, 2014, to January 30, 2020, for the discovery and validation sets.

The discovery set of patients, recruited between March 2014 and September 2016, comprised 63 pregnant participants (Figure 1, Table 1, and Supplemental Table 1; supplemental material available online with this article; https://doi.org/10.1172/JCI169199DS1). Follow-up for the ascertainment of study outcomes was completed in November 2016. The validation set of patients, recruited between October 2016 and January 2020, comprised 60 pregnant participants, with follow-up for the ascertainment of study outcomes completed in March 2020. There were no significant differences in maternal demographics, pregnancy characteristics, or pregnancy outcomes between the discovery and validation sets (Table 1, Supplemental Table 2, and Supplemental Figure 1). Overall, 42 (34%) of the pregnancies ended in the primary outcome of fetal or neonatal death (within the first 28 days of life). For the 3 secondary outcomes, only fetal death or delivery at or before 28+0 weeks of gestation could be ascertained for all pregnancies, occurring in 58 (47%) of them. The UmA Doppler velocimetry was normal (≤95th centile for gestation; ref. 28) at enrollment in 46 participants, of whom 21 (46%) subsequently developed abnormal UmA Doppler measurements. Fetal growth trajectory (based on the change in percentage of weight deviation over a period or 2 weeks or more) (29) could be assessed for 104 pregnancies (85%), with the remaining pregnancies ending in fetal death or delivery before a 2-week interval was reached. Forty-one of these 104 fetuses (39%) demonstrated slow fetal growth (worsening of weight deviation of 10 percentage points). A smaller proportion of fetuses demonstrated slow fetal growth in the validation set (31%) than in the discovery set (47%).

### Ultrasound measurements as predictors of fetal or neonatal death and death or delivery at or before 28+0 weeks of gestation in the discovery set.

The best ultrasound predictor of fetal or neonatal death was the EFW *z* score, either as calculated using the Hadlock 3 formula and the Marsal chart (EFW-HM: AUC, 0.81; 95% CI, 0.69–0.93) or the Intergrowth formula and chart (EFW-Int: AUC, 0.83; 95% CI, 0.71–0.95). UmA category (≤95th centile; >95th centile with positive end-diastolic flow [EDF]; absent EDF; reversed EDF; AUC, 0.75; 95% CI, 0.62–0.88) and slow fetal growth (AUC, 0.70; 95% CI, 0.56–0.83) were also fair predictors. The UmA category was the best predictor of death or delivery at or before 28+0 weeks (AUC, 0.80; 95% CI, 0.70–0.91), with the mean UtA pulsatility index (PI) (AUC, 0.77; 95% CI, 0.65–0.89) and the EFW-Int *z* score (AUC, 0.73; 95% CI, 0.60–0.85) also fair predictors (Supplemental Tables 3 and 4).

### Proteomics.

Mass spectrometry (MS) profiling of pooled samples gave quantitative information for 200 protein groups (sets of proteins that cannot be distinguished on the basis of peptide sequences), from which chorionic somatomammotropin hormone (CSH, also known as human placental lactogen), fibronectin, pregnancy-specific β-1 glycoprotein 1 (PSG1), serum amyloid A (SAA), and leucyl-cystinyl aminopeptidase (LNPEP) were selected for individual validation on the basis of the scoring system outlined in Methods.

### Univariate associations between maternal serum protein concentrations and outcomes in the discovery set.

**Figure 2:**
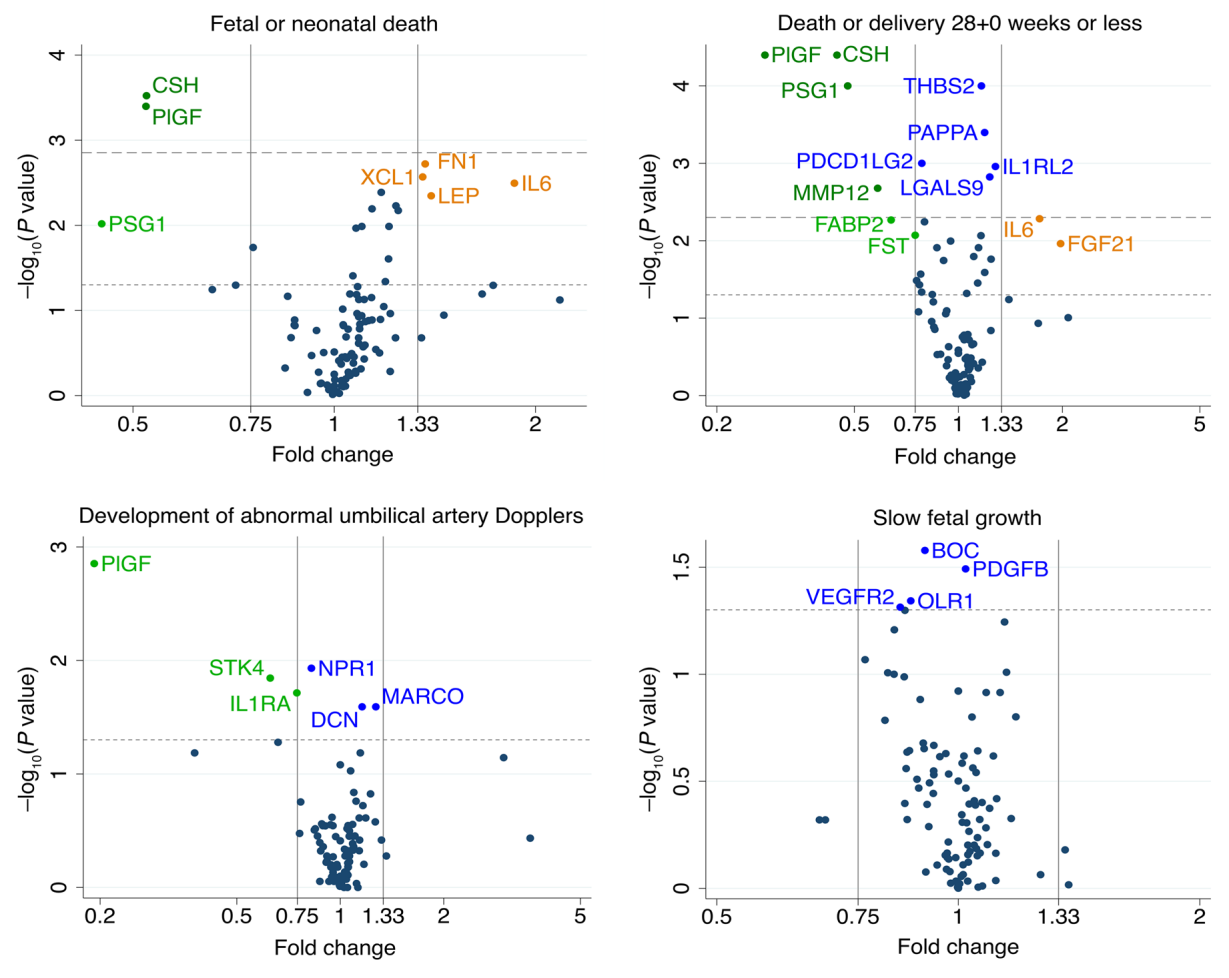
Volcano plots showing the statistical significance and magnitude of the associations between the 98 proteins and 4 pregnancy outcomes in the discovery set. Associations were tested with a 2-sided *t* test for symmetrical data and a Mann-Whitney *U* test for skewed data. Dotted line indicates a *P* value of 0.05. Dashed line indicates the Benjamini-Hochberg cutoff with a 5% FDR (*P* = 0.0015 for fetal or neonatal death, *P* = 0.005 for death or delivery ≤28+0 weeks). See Supplemental Table 5 for full protein names.

Four proteins were undetectable in most of the samples: VEGFA, natriuretic peptides B (BNP), melusin, and poly[ADP-ribose] polymerase 1 (PARP1). These were excluded from further prediction analyses. The associations between the remaining 98 proteins and the 4 pregnancy outcomes are summarized in Figure 2. Placental growth factor (PlGF) and CSH concentration were significantly associated with fetal or neonatal death (after Benjamini-Hochberg correction), with fold changes of 0.52 in pregnancies ending in fetal or neonatal death compared with pregnancies ending in neonatal survival. The concentrations of 9 proteins were significantly associated with death or delivery at or before 28+0 weeks (after correction). The greatest magnitudes of fold changes were seen for PlGF (0.28), CSH (0.45), and PSG1 (0.48).

### Parenclitic network analysis of the discovery set.

**Figure 3:**
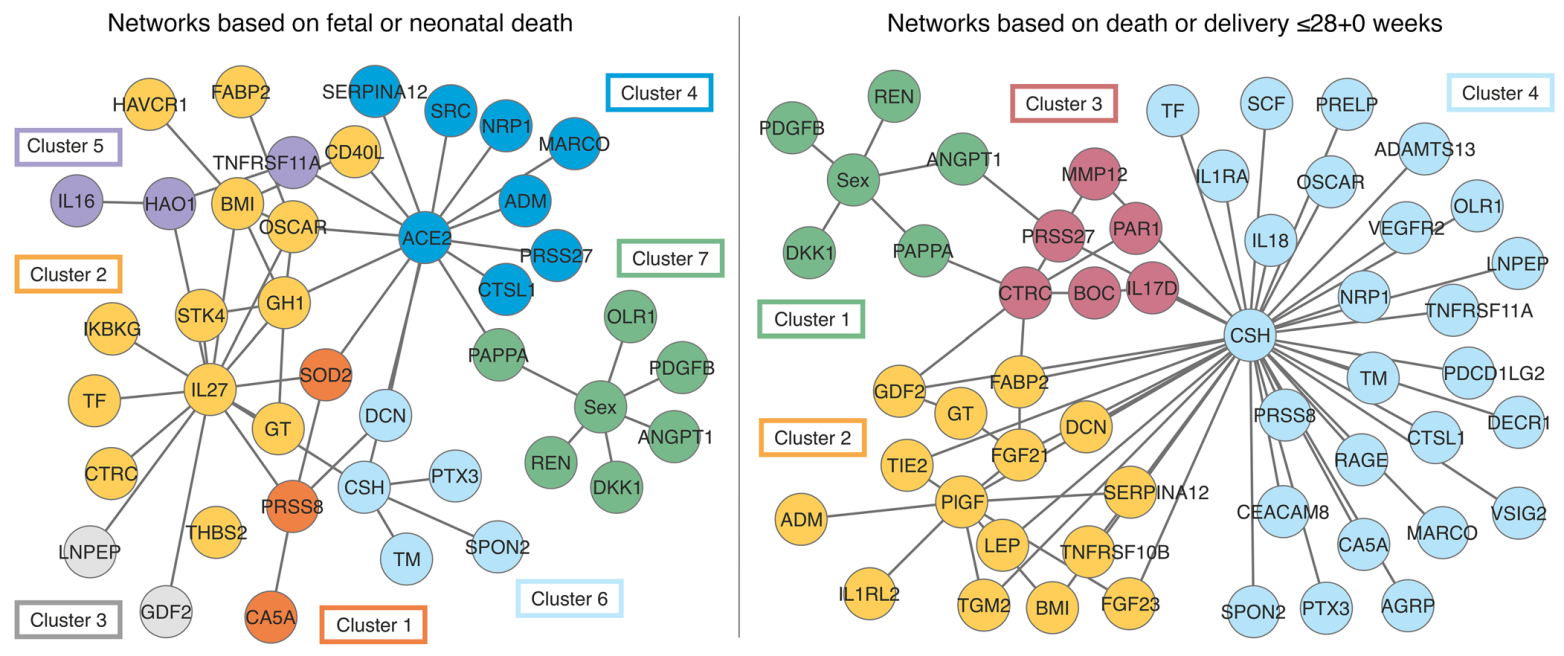
Parenclitic network analysis and clustering by pregnancy outcome. Networks were generated on the basis of pregnancies ending in fetal or neonatal death versus live births surviving to 29 days of life and pregnancies ending in fetal death or delivery at or before 28+0 weeks of gestation versus continuation of pregnancy beyond 28+0 weeks. See Supplemental Table 5 for full protein names and Supplemental Figures 2 and 3 for the associated dendrograms.

Both the networks for fetal or neonatal death and death or delivery at or before 28+0 weeks contained clusters centered around CSH (clusters 6 and 4, Figure 3). These clusters also contained the pentraxin-related protein PTX3, spondin 2 (SPON2), and thrombomodulin (TM) and contained or were linked to decorin (DCN). The network for death or delivery at or before 28+0 weeks also contained a cluster centered around PlGF (cluster 2). For all 3 of the networks that included fetal sex, similar clusters emerged that contained renin (REN), angiopoietin 1 (ANG1), dickkopf-related protein 1 (DKK1), and platelet-derived growth factor subunit β (PDGFβ), that contained or were linked to pregnancy-associated plasma protein A/pappalysin 1 (PAPPA) and in 2 of the 3 networks included oxidized low-density lipoprotein receptor 1 (OLR1). Networks and associated dendrograms for the development of abnormal UmA Dopplers and slow fetal growth are provided in Supplemental Figures 4 and 5.

### Model selection by stakeholders.

**Figure 4:**
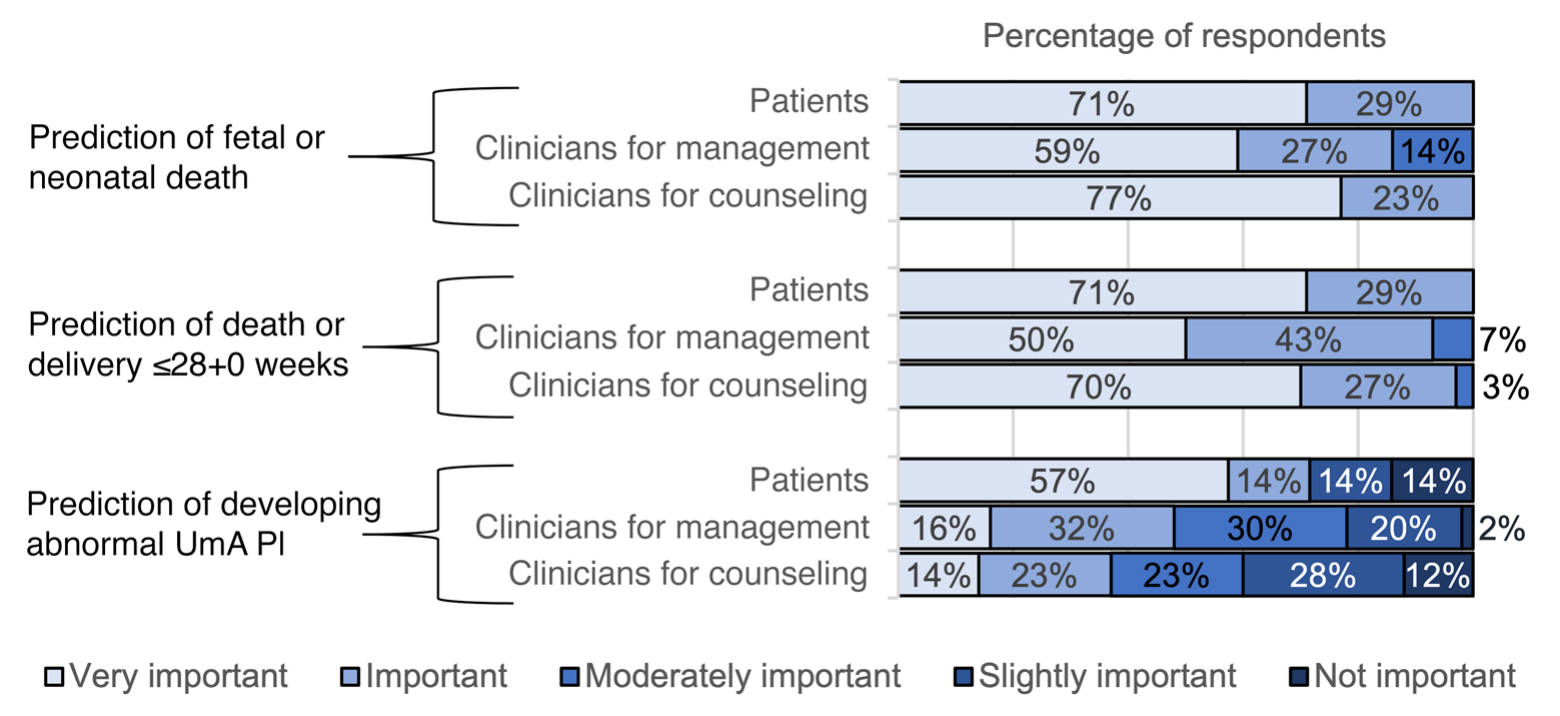
The perceived importance to patients and clinicians of our 3 pregnancy outcomes for model validation. Importance ranked on a 5-point Likert scale with clinicians asked to separately judge the importance for pregnancy management and the importance for patient counselling.

None of the single-variable or multivariable models for predicting slow fetal growth performed well enough to warrant validation (AUCs, <0.70). For the 3 remaining outcomes, an online survey was performed to ascertain the priorities of clinicians and patients in predicting outcomes. Forty-five clinicians from 18 countries (of 173 contacted, 26%) and 7 patients from the United Kingdom who had experienced a pregnancy complicated by severe, early-onset FGR (of 36 contacted, 19%) responded (Supplemental Table 6). The prediction of fetal or neonatal death and death or delivery at or before 28+0 weeks were considered important or very important by all patients and were also rated highly by clinicians for the purposes of patient counseling and clinical management (Figure 4). Patients and clinicians marginally prioritized sensitivity over specificity for most outcomes (Supplemental Figure 6). For the prediction of the development of abnormal UmA PI, patients universally prioritized sensitivity, whereas clinicians marginally prioritized specificity for patient counseling.

Based on the survey results, the model performance metrics, and the assay reliability, models containing the variables listed in Tables 2 and 3 were selected for validation. For the prediction of death or delivery at or before 28+0 weeks, models including CSH marginally outperformed models including PlGF. However, possibly because of the short processing time, the commercial CSH ELISA had high intra-assay variability in our hands (mean coefficient of variation, 8.0%; SD, 7.3%; 28% requiring repeat analysis for coefficient of variation >10%). Because of this and the existence of clinically approved tests for PlGF, models including PlGF were selected for validation.

### Model validation.

Five of the 7 protein models (Table 2) and all 5 of the models containing ultrasound measurements (Table 3), generated in the discovery set, were successfully validated, with AUCs included in the AUC 95% CIs generated from the discovery cross-validation estimates.

### Addition of pregnancy characteristics.

Validated models were not significantly improved by the addition of maternal BMI, maternal age, maternal ethnicity, or fetal sex. Adding gestational age at enrollment significantly improved the models containing EFW-HM alone (likelihood ratio [LR] test, *P* = 0.0001) and EFW-HM with the UmA category (LR test, *P* < 0.00005) to predict fetal or neonatal death (Supplemental Table 7). The addition of “preeclampsia at enrollment” significantly improved all validated models predicting fetal death or delivery at or before 28 weeks’ gestation (Supplemental Table 7).

### PlGF values for maximum LRs.

**Figure 5:**
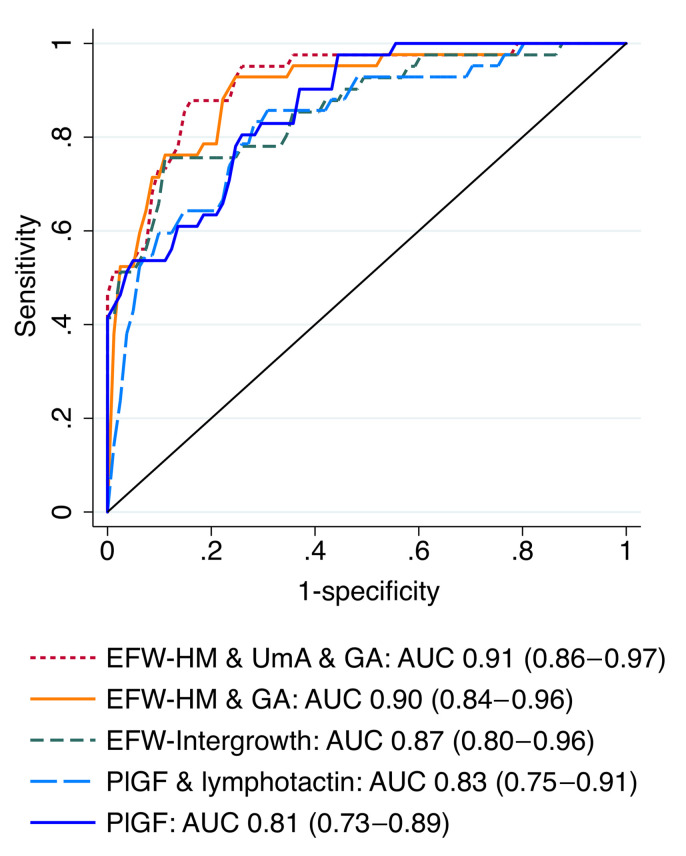
Comparison of the ROC curves for the models predicting fetal or neonatal death. EFW-HM, estimated fetal weight calculated using the Hadlock 3 formula (67), with the *z* score calculated using the Marsal reference chart (68); EFW-Intergrowth, estimated fetal weight and *z* score calculated using the Intergrowth formula and reference chart (69); GA, gestational age at enrollment; PlGF, placental growth factor concentration. UmA Doppler category: 0 = PI ≤95th centile, 1 = PI >95th centile, 2 = absent EDF, 3 = reversed EDF.

**Figure 6:**
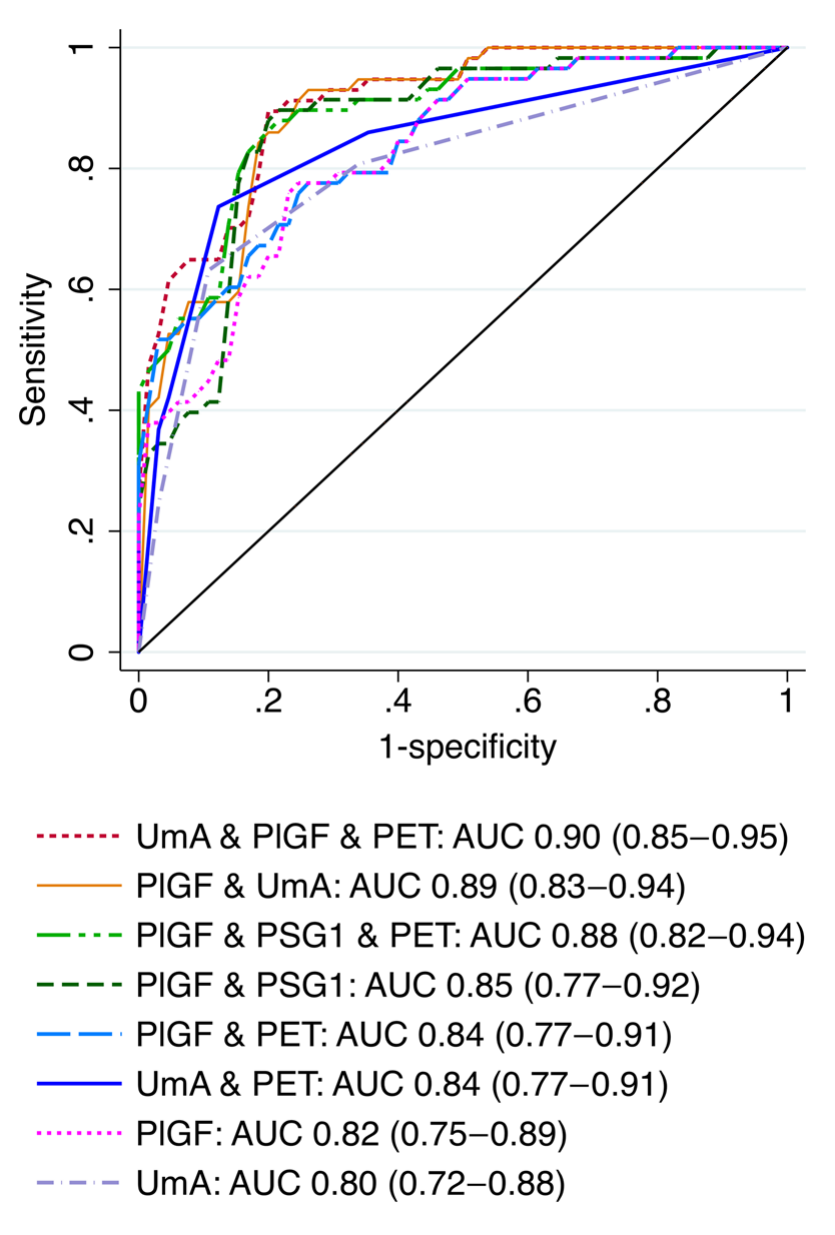
Comparison of the ROC curves for the models predicting fetal death or delivery at or before 28+0 weeks’ gestation. PET, preeclampsia at enrollment; PSG1 NPX. UmA Doppler category: 0 = PI ≤95th centile, 1 = PI >95th centile, 2 = absent EDF; 3 = reversed EDF.

Receiver operating characteristic (ROC) curves are shown in Figures 5 and 6. Model constants and coefficients, along with optimal cut points for positive and negative LRs and correct classification are provided in Supplemental Tables 8–10. A serum PlGF concentration below 14.2 pg/mL predicted fetal or neonatal death with a positive LR of 18.3, a sensitivity of 45%, and a specificity of 98% and correctly classified 80% of participants. A serum PlGF concentration below 14.5 pg/mL predicted death or delivery at or before 28+0 weeks with a positive LR of 24.7, a sensitivity of 38%, and a specificity of 98% and correctly classified 70% of participants.

### Alternative EFW formulas.

Although the EFW *z* score calculated using the Intergrowth formula and chart gave the highest AUC for predicting fetal or neonatal death, the Intergrowth formula for estimating fetal weight performed poorly in our sample, especially at lower fetal weights. For the 21 live-born neonates with a birth weight of less than 600 g and an EFW performed within 7 days of delivery, the Intergrowth formula overestimated birth weight by a mean of 47% (SD, 14%), in contrast to the Hadlock 3 formula, which overestimated birth weight by a mean of 25% (SD, 10%; Supplemental Figure 8). For all 67 live births of infants with an EFW performed within 7 days of delivery, the Intergrowth formula overestimated birth weight by a mean of 29% (SD, 20%), and the Hadlock 3 formula overestimated birth weight by a mean of 15% (SD, 13%). As might be expected, use of the EFW calculated from 1 formula in the model derived from the other had a substantially negative impact on calibration (Supplemental Figure 9).

### Reanalysis of the combined sets.

**Figure 7:**
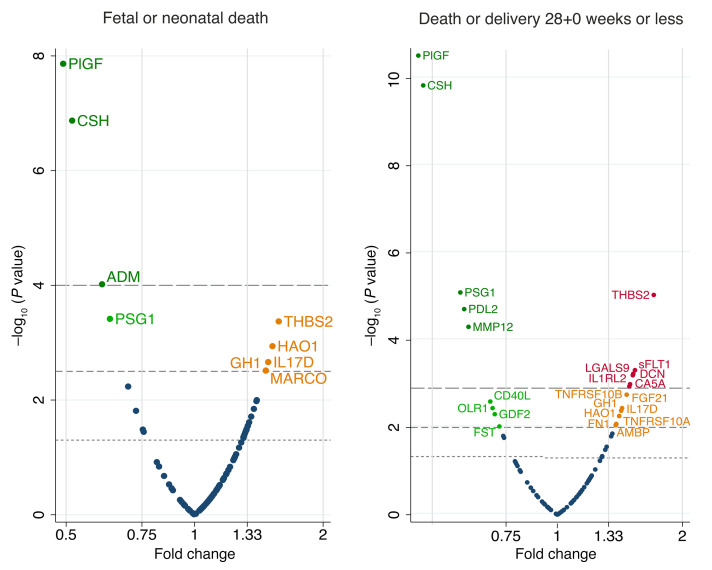
Volcano plots showing the statistical significance and magnitude of associations between pregnancy outcome and the centered and scaled concentrations of the 93 proteins from the discovery and validation sets combined. Associations were tested with 2-sided *t* tests. The dotted line indicates a *P* value of 0.05; the short-dashed line indicates the Benjamini-Hochberg cutoff with a 5% FDR (*P* = 0.0048 for fetal or neonatal death, *P* = 0.012 for death or delivery ≤28+0 weeks); and the long-dashed line indicates the Benjamini-Hochberg cutoff with a 1% FDR (*P* = 0.00032 for fetal or neonatal death, *P* = 0.0013 for death or delivery ≤28+0 weeks). See Supplemental Table 10 for full protein names and individual –log_10_ *P* values.

Combining the centered and scaled data from the discovery and validation sets, the strongest associations with both fetal or neonatal death and death or delivery at or before 28+0 weeks were the previously observed negative associations with PlGF (*P* = 1.4 × 10^–8^ and *P* = 3.0 × 10^–11^) and CSH (*P* = 1.3 × 10^–7^ and *P* = 1.4 × 10^–10^) (Figure 7). The evidence for the negative association between PSG1 and both outcomes was strengthened, as was the evidence for negative associations between matrix metalloproteinase 12 (MMP12) and programmed cell death 1 ligand 2 (PDCD1LG2) and death or delivery at or before 28+0 weeks. None of the proteins showed an association with the development of abnormal UmA Dopplers or slow fetal growth at a Benjamini-Hochberg 5% FDR (Supplemental Figure 10).

**Figure 8:**
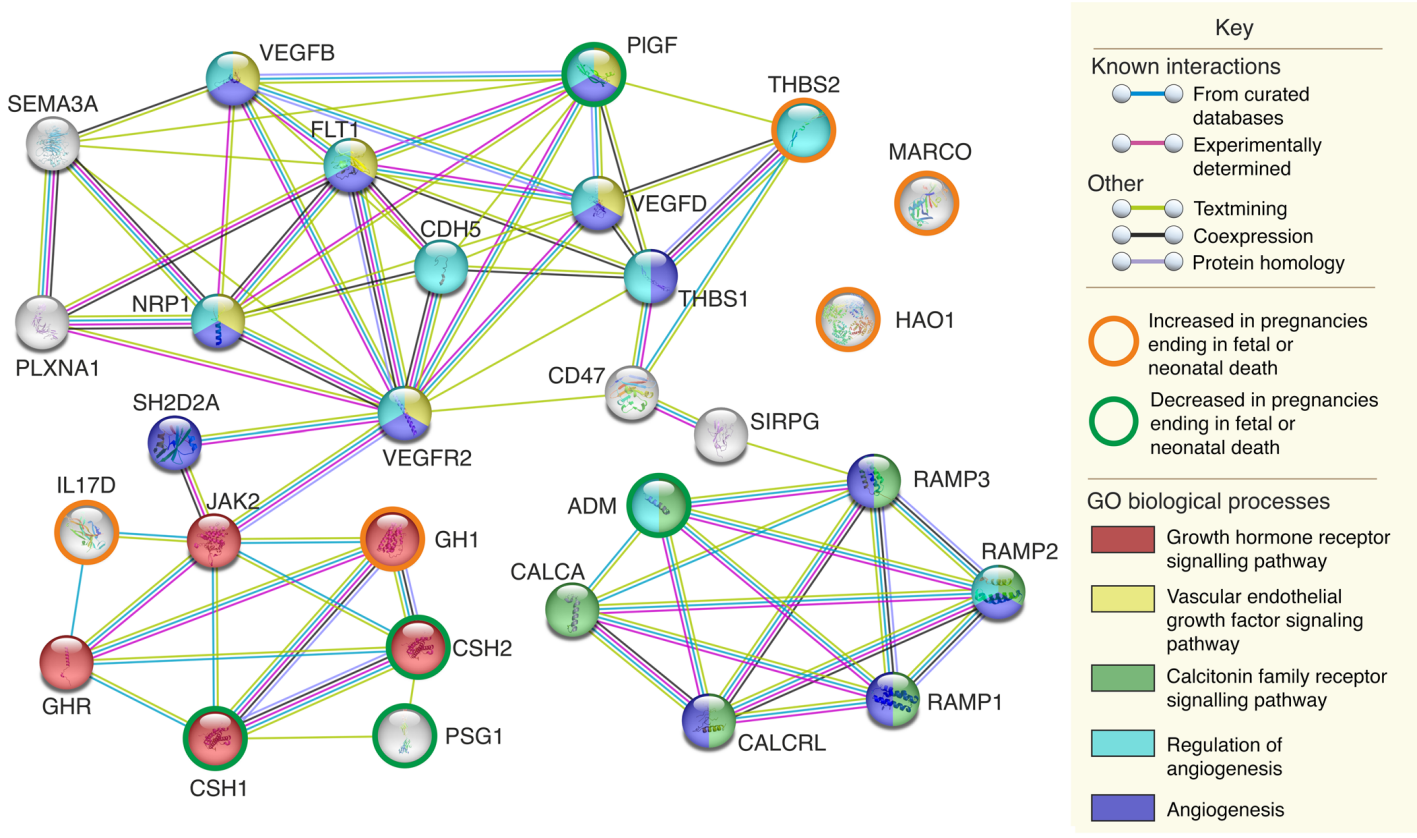
An expanded functional network demonstrating interactions and shared GO biological processes of the proteins associated with fetal or neonatal death in the combined data set at a Benjamini-Hochberg FDR of 5%. See Supplemental Table 5 for full protein names. The analysis, graphics, and legend are from the Swiss Institute of Bioinformatics (STRING) (81).

**Figure 9:**
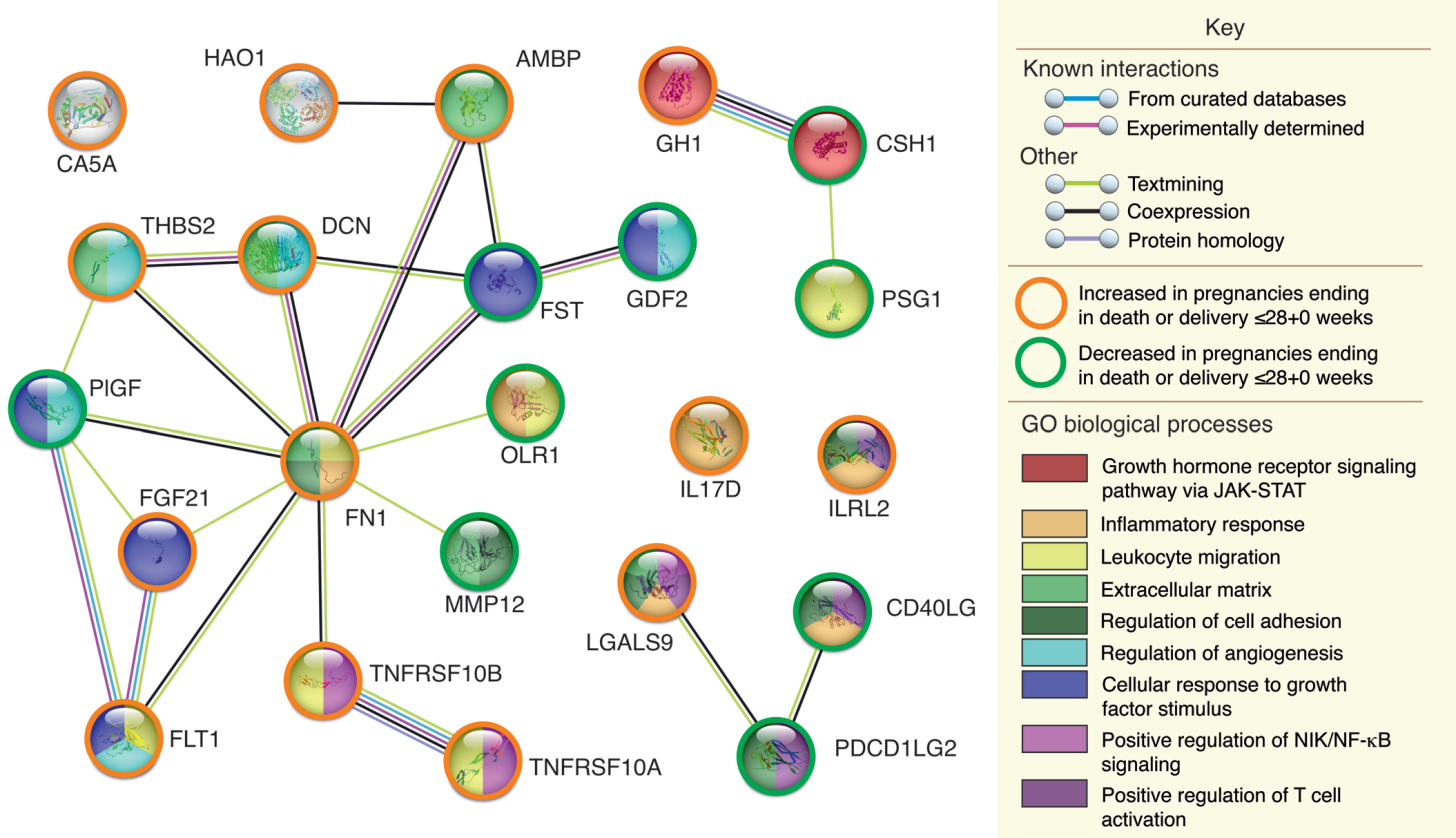
Functional interactions and shared GO biological processes of the proteins associated with death or delivery at or before 28+0 weeks in the combined data set at a Benjamini-Hochberg FDR of 5%. See Supplemental Table 5 for full protein names. The analysis, graphics, and legend are from STRING (81).

Functional analysis of the proteins associated with fetal or neonatal death at a 5% FDR demonstrated coexpression of CSH and growth hormone (GH). Expanding the network to include intervening proteins resulted in clusters sharing Gene Ontology (GO) biological processes of GH receptor signaling, VEGF signaling, and calcitonin family receptor signaling, with proteins in the latter 2 clusters also involved in angiogenesis and regulation of angiogenesis (Figure 8). Proteins associated with death or delivery at or before 28+0 weeks showed multiple interactions that were predominantly centered on fibronectin. Shared GO biological processes included those relating to growth (regulation of angiogenesis, cellular response to growth factors, and the GH receptor signaling pathway via JAK/STAT), immune function (leukocyte migration, inflammatory response, and positive regulation of T cell activation), or both (regulation of cell adhesion, positive regulation of NIK/NF-κB signaling) (Figure 9).

The 3 best-performing leave-one-out cross-validation (LOOCV) models using the combined centered and scaled data all included pro-adrenomedullin (ADM) for predicting fetal or neonatal death; PlGF and CSH for predicting death or delivery at or before 28+0 weeks; and PDGFB for predicting the development of abnormal UmA Doppler measurements (Supplemental Table 12). The emergence of ADM in the models predicting fetal or neonatal death was consistent with the significant association present in the combined (*P* = 0.0001), but not discovery (*P* = 0.15), sets. In contrast, PDGFβ did not show significant univariate associations with the development of abnormal UmA Dopplers in the discovery, validation, or combined data sets.

### Predicting gestational age of a live-birth infant or diagnosis of fetal death and interval from enrollment to live birth or diagnosis of fetal death.

**Figure 10:**
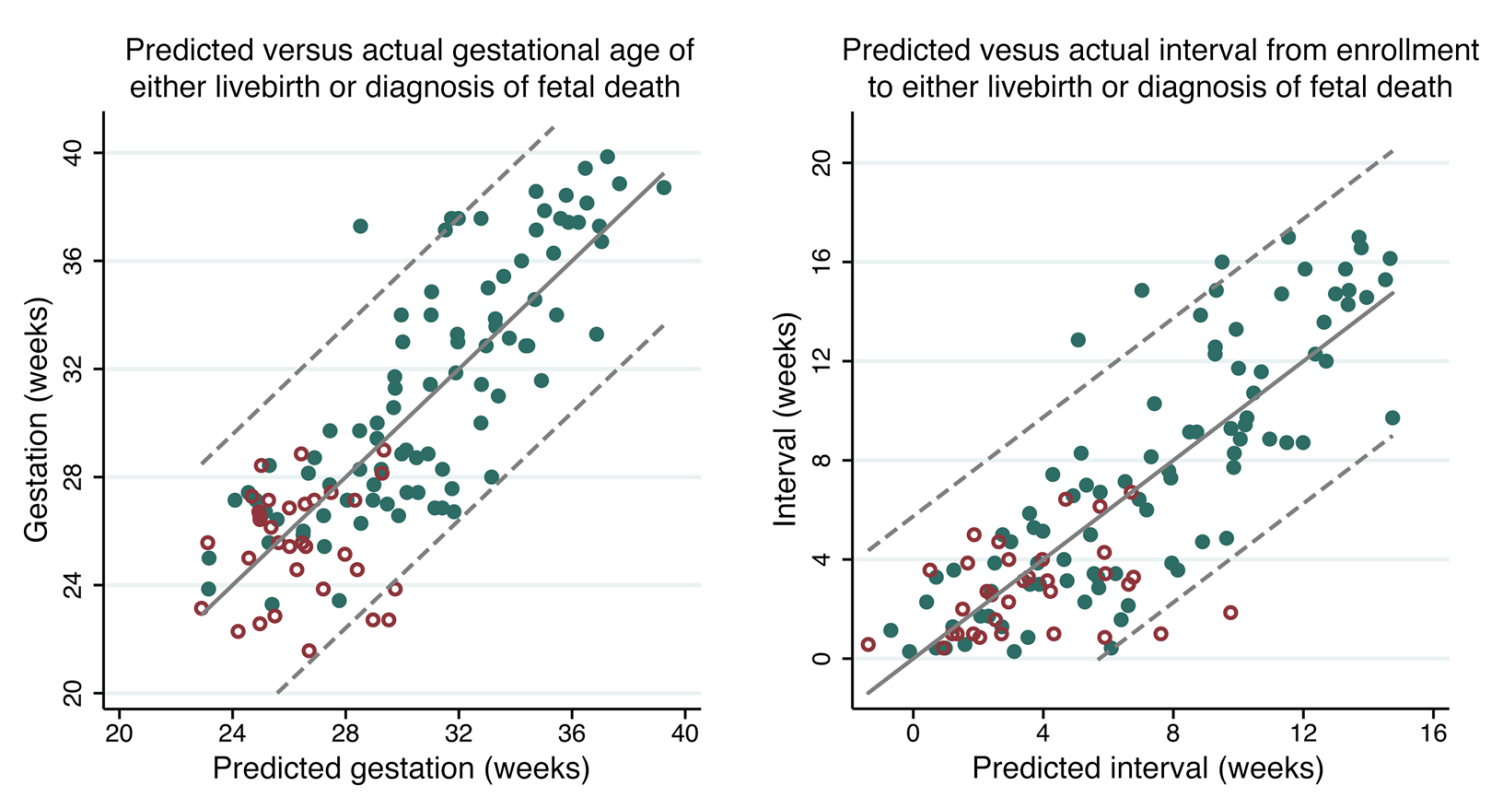
Observed versus predicted value graphs for models predicting the timing of live birth or fetal death. Model predicting either the GA of a live-born neonate or the diagnosis of fetal death includes PlGF and sFLT1 concentrations, decorin and matrix metalloproteinase 12 NPX, and UmA Doppler category. Model predicting the interval from enrollment to either live birth or the diagnosis of fetal death includes PlGF and sFLT1 concentrations, decorin and matrix metalloproteinase 12 NPX, UmA Doppler category, and GA at enrollment. Solid green circles indicate pregnancies ending in a live birth; red hollow circles indicate pregnancies ending in fetal death; dotted lines indicate 95% prediction intervals.

Twelve protein and ultrasound measurements showed an association with the gestational age at which the pregnancies ended in either a live birth or fetal death, at a 1% Benjamini-Hochberg FDR (Supplemental Table 13). The best model to predict gestational age at live birth or fetal death included PlGF and soluble FMS-like tyrosine kinase 1 (sFLT1) concentrations, MMP12 and IL-1 receptor-like 2 (IL-1RL2) normalized protein expression (NPX), and UmA category at enrollment (Figure 10). Eight protein and ultrasound measurements showed an association with the interval between enrollment and either live birth or the diagnosis of fetal death, at a 1% Benjamini-Hochberg FDR (Supplemental Table 13). The best model to predict the interval between enrollment and live birth or fetal death included PlGF and sFLT1 concentrations, MMP12 and decorin NPX, UmA category, and gestational age at enrollment (Figure 10). Both models accounted for 68% of the variation in the outcomes they were predicting but had 95% prediction intervals of 40 days, limiting their clinical utility. Sparser models, including PlGF and sFLT1 concentrations and UmA category to predict gestational age at live birth or fetal death, and these same variables plus gestational age at enrollment to predict interval to live birth or fetal death, had only slightly wider 95% prediction intervals of 42 days (Supplemental Figure 11).

### Placental histological classification.

Placental samples for histological examination were available for 55 pregnancies (45%); these had characteristics and outcomes similar to those of the pregnancies without available samples (Supplemental Tables 14 and 15). The only statistically significant difference was a higher proportion of female fetuses among the pregnancies that had placental samples than those that did not (63% vs. 41%, *P* = 0.016). Forty-five (82%) placentas showed evidence of placental pathology, with 39 (71%) classified as maternal vascular malperfusion (MVM), 3 (5%) as villitis of unknown etiology (VUE), 1 (2%) as fetal vascular malperfusion (FVM), and 2 (4%) as nonspecific dysmorphic villi. Twelve of the 14 placental samples from pregnancies ending in stillbirth showed MVM, whereas the 3 available samples from pregnancies ending in neonatal death showed VUE, FVM, and dysmorphic villi. Mean UtA PI (*P* = 0.044), maternal serum PlGF (*P* = 0.043), and maternal serum PAPPA (*P* = 0.036) at enrollment all differed significantly between pregnancies with subsequent MVM and pregnancies without MVM. In contrast, none of the UmA parameters studied (UmA category at enrollment, the occurrence of UmA PI above the 95th centile at any point before delivery, and the occurrence of absent or reversed UmA EDF at any point before delivery) showed evidence of an association with placental histological classification of MVM (Supplemental Table 16).

## Discussion

### Principle findings and significance.

To our knowledge, this is the first study to use a discovery science approach, combining ultrasound and biochemical parameters, to identify and validate prognostic markers at the time of diagnosis of severe, early-onset FGR. These findings can be used to inform personalized counseling and management of affected pregnancies with outcomes of importance to patients and clinicians. Furthermore, by providing alternative thresholds that prioritize positive and negative LRs and maximum correct categorization, eligibility criteria for clinical trials of novel therapeutics can be adapted depending on the perceived risk/benefit ratio of the intervention.

Our secondary analyses, including parenclitic network analysis, functional enrichment analysis, and triangulation with placental histological classification, provide a deeper characterization of this unique case series. Some of these findings support and enhance our existing understanding of placental FGR, such as the interplay between angiogenesis, immune cells, and the extracellular matrix (30–32). Other findings from our study offer new avenues for investigation, such as the parenclitic network cluster around fetal sex, which includes proteins related to pericyte function (33).

### Findings in the context of the existing literature.

Given that ultrasound assessment of biometry and Doppler velocimetry forms the mainstay of identification and monitoring of FGR, it is unsurprising that the EFW *z* score and UmA category were validated as predictors of fetal or neonatal death and fetal death or delivery at or before 28+0 weeks, respectively (34–36). A secondary analysis of 105 pregnancies from the UK placebo-controlled trial of sildenafil citrate for early-onset FGR (STRIDER) (EFW or abdominal circumference [AC] <10th centile with absent or reversed UmA EDF at 22+0 to 29+6 weeks) identified EFW as an independent predictor of live birth (OR per 100 g, 4.3; 95% CI, 2.3–8.0; *P* < 0.001) and overall survival (OR per 100 g, 2.9; 95% CI, 1.8–4.4; *P* < 0.001) (37). What is less expected is that absent or reversed ductus venosus (DV) a-wave was a poor predictor of fetal or neonatal death in our participants (AUC, 0.59, 95% CI, 0.53–0.66; see Supplemental Table 4), in contrast to the results of previous studies (38, 39). This may reflect a change in clinical practice since the publication of these studies. Their findings led to the DV waveform becoming an important factor in the timing of delivery in extremely preterm FGR (14, 40), which may have altered the natural history of the disease by prompting delivery before stillbirth could occur.

A limitation of using ultrasound parameters is their potential for variation. In the case of Doppler velocimetry, this includes interobserver variability, temporal variation due to factors such as maternal and fetal movement, and variation in UmA waveforms between arteries and along the length of the cord (41–44). There is also considerable variation between different Doppler reference ranges, both in terms of the values of their “normal” ranges and their methodological quality (45). In the case of the EFW *z* score, variation arises from interobserver variability in measuring biometry, variation in formulas used to generate the EFW, and variation in charts used to determine the *z* score for gestational age (46–48). Despite the methodological limitations of the Hadlock 3 formula, a recent study of 65 pregnancies with early-onset FGR in which the woman delivered within 7 days of ultrasound assessment found that it gave a better combination of systematic and random error than the 20 other formulas tested (49).

Several recent studies have highlighted the potential utility of PlGF concentration to predict outcomes in small-for-gestational-age (SGA) and FGR pregnancies. In a case series of 173 singleton pregnancies with a customized EFW below the tenth centile between 20+0 and 31+6 weeks, the sFLT1/PlGF ratio at diagnosis was an excellent predictor of delivery before 30 weeks (AUC, 0.96; 95% CI, 0.93 to <0.99) and before 34 weeks (AUC, 0.94; 95% CI, 0.89–0.98) and a good predictor of a composite adverse perinatal outcome (AUC, 0.83; 95% CI, 0.77–0.90) (50). Similarly, in 116 singleton pregnancies with early-onset FGR (customized EFW below the third centile or customized EFW below the tenth centile with abnormal UmA and/or UtA Doppler velocimetry; <32+0 weeks) and positive UmA EDF ending in live birth, women with an sFLT1/PlGF ratio of 85 or higher were significantly more likely to deliver within 1, 2, 3, and 4 weeks from the ratio measurement than were women with an a sFLT1/PlGF ratio of less than 85 (51). Composite neonatal morbidity and neonatal admission were also significantly higher following pregnancies with a sFLT1/PlGF ratio of 85 or higher (53.8% vs. 28.6%, *P* = 0.04; 97.5% vs. 67.9%, *P* < 0.01). More strikingly, in a series of 130 singleton pregnancies with SGA (AC or EFW, below the tenth centile), fetal demise only occurred in pregnancies with a PlGF below the tenth centile for gestational age at any time between 16 and 36 weeks (12 of 65 vs. 0 of 65, *P* < 0.0001) (52).

While these studies revealed the PlGF results to the managing clinicians, similar results have been found in studies in which PlGF was not revealed. The secondary analysis of the STRIDER UK trial participants, mentioned above, found significant associations between pregnancy outcomes and both the sFLT1/PlGF ratio and PlGF alone (37). Higher PlGF concentrations and lower sFLT1/PlGF ratios were associated with better overall survival (PlGF coefficient, 3.67, *P* < 0.001; ratio coefficient, 0.51, *P* = 0.002) and later gestation at birth (PlGF coefficient, 1.4, *P* < 0.001; ratio coefficient, –0.99, *P* < 0.001). Similarly, in a multinational case series of 411 pregnancies, PlGF concentration below the fifth centile at the time of suspected FGR (AC below the tenth centile from 20+0 weeks) had 87.5% sensitivity and 62.8% specificity for predicting stillbirth (53). A PlGF concentration below 12 pg/mL was associated with a shorter interval to delivery than a PlGF concentration above the fifth centile (13.0 vs. 29.5 days, *P* < 0.0001).

Our finding that the placental histological classification of maternal vascular malperfusion was significantly associated with a lower maternal PlGF concentration and a higher mean UtA PI at the time of diagnosis of early-onset FGR, but not with UmA Doppler measurements, was in keeping with the results of previous studies (53–56). Agrawal et al. recently reported that MVM, unlike other placental pathologies, is characterized by an increase in the mean UtA PI and a gradual decline in the PlGF concentration as the pregnancy progresses (54). Triunfo et al. found in SGA pregnancies (EFW <10th centile) identified between 30 and 34 weeks of gestation a pattern of placental histopathology they termed “placental underperfusion,” was most strongly associated with lower PlGF, measured at the time of diagnosis (55). Benton et al. also found a low PlGF concentration to be a better predictor of placental pathology than the UmA resistance index or the abdominal circumference centile (53).

### Strengths and limitations.

The strengths of this multicenter study are that it was carried out prospectively in academic health science centers with fetal medicine experts trained according to a common ultrasound protocol and level 3 perinatal care available for delivery. Participants and their fetuses/neonates were extensively phenotyped at study entry, for the duration of the pregnancy, and postnatally, and we report temporally validated results. All pregnancies were managed according to local guidelines that were broadly consistent and in line with national and international guidelines (4, 16, 57, 58) as well as current randomized, controlled trial (RCT) evidence (e.g. the TRUFFLE trial). This introduces variation, but potentially better reflects real-world practice and hence adds external validity. All serum analysis was carried out after pregnancy outcomes were obtained using a proteomics discovery science approach that did not assume associations with outcome but that also included additional analysis of proteins anticipated to be related to pregnancy outcome in placental insufficiency. Placental histological classification was blinded to pregnancy outcomes and included control and non-FGR preterm placental samples to remove some potential bias. Finally, our inclusion of stakeholders to guide model selection means that their predictive value is most important to patients and clinicians.

Our relatively narrow inclusion criteria are both a strength and a limitation, in that they allowed us to focus on a specific clinical group but limited our sample size and the generalizability of our findings. The sample size means our study was underpowered to demonstrate small or medium effects, and our estimates have wider CIs than those for larger studies (59). The exclusion of pregnancies below the third centile but above an EFW of 600 g limits the number of pregnancies from 24+6 weeks of gestation to which our findings can be applied, and the exclusion of pregnancies with known genetic, chromosomal, and structural differences means our findings cannot be applied to the whole spectrum of FGR. Generalizability is also limited to health care settings with comparable neonatal care provision and outcomes, given their impact on neonatal survival and decision making for iatrogenic preterm delivery. Clinicians managing the pregnancies were not blinded to ultrasound measurements, and, indeed, many management decisions will have been influenced by the ultrasound findings. This could have biased the apparent associations between ultrasound variables and pregnancy outcomes, either artificially strengthening or weakening them.

### Future directions.

Ideally, our findings should be independently and externally validated. Given the incidence of FGR at or before 28+0 weeks, this would require another multicenter study. Further research is also needed to determine whether the use of these models would have benefit in practice, both on the psychological well-being of parents and on the use of health resources. Future studies to identify and validate predictive models in early-onset SGA (EFW below the tenth centile, before 32+0 weeks of gestation) would allow application to a wider population. This would complement the work currently being done in the PLANES (placental growth factor led management of the small for gestational age fetus) study, which is investigating the effect of revealed PlGF in SGA from 32+0 weeks (60). Finally, our primary outcome of fetal or neonatal death provides only short-term information, and data collection for 2-year neurodevelopmental outcomes is ongoing.

### Conclusion.

In conclusion, our study provides validated models for predicting fetal or neonatal death and fetal death or delivery at or before 28+0 weeks of gestation based on ultrasound and maternal serum protein measurements at the time of diagnosis of severe, early-onset FGR. The EFW *z* score and UmA Doppler velocimetry were the best-performing ultrasound parameters, but are vulnerable to inter-rater variability, variation in formulas and reference ranges, and temporal variation. The biomarker PlGF was the best-performing maternal serum protein for predicting both pregnancy outcomes and MVM. This identification of a specific pathological phenotype may be useful for targeting future potential therapies.

## Methods

Additional details can be found in the Supplemental Methods.

This study is reported according to the STrengthening the Reporting of OBservational studies in Epidemiology (STROBE) guidelines (61) for cohort studies and the transparent reporting of a multivariable prediction model for individual prognosis or diagnosis (TRIPOD) guidelines (62).

### Study design and setting.

The EVERREST prospective study was a multicenter prospective cohort study recruiting pregnant women from 4 European tertiary referral centers: University College London Hospital, United Kingdom; University Medical Centre Hamburg-Eppendorf, Germany; Maternal-Fetal Unit Hospital Clinic, Barcelona, Spain; and Skane University Hospital, Lund, Sweden.

### Study population.

Full details on the protocol have been published previously (26). In brief, pregnant women were eligible if they had a singleton fetus with an ultrasound EFW below 600 g and below the third centile according to local criteria between 20+0 and 26+6 weeks of gestation. Exclusion criteria included a known abnormal karyotype or a major fetal structural abnormality at enrollment (63); indication for immediate delivery; preterm rupture of membranes before enrollment; maternal HIV or hepatitis B or C infection (because of the impact on processing and storage of biological samples); maternal age under 18 years; any medical or psychiatric condition that compromised the woman’s ability to participate; and a lack of capacity to consent. Pregnant women with a known congenital infection were not recruited, and for the purposes of this analysis, pregnancies that were terminated were excluded. Decisions to terminate (*n* = 7) were based on parental concerns about the short- and long-term prognosis for the fetus and maternal health risks.

### Outcomes.

The primary outcome was fetal or neonatal death (≤28 days of life). Secondary outcomes were: fetal death or delivery at or before 28+0 weeks of gestation; slow fetal growth, defined as a worsening of weight deviation of 10 or more percentage points over a 2-week interval (including before and after enrollment) or an equivalent trajectory over a longer period (29); and the development of abnormal UmA Dopplers, defined as development of the UmA PI above the 95th centile in pregnancies in which the UmA PI was at 95th centile or below at enrollment (28). Slow fetal growth was selected as a secondary outcome because it showed an association with fetal or neonatal death in the discovery set but could only be assessed with serial scans. A surrogate biomarker could potentially give the same information at the time of diagnosis and provide pathophysiological insights. Ascertainment for outcomes of this study was possible, at the latest, by 29 days of life. Follow-up for neonatal morbidity and infant health and neurodevelopment to the age of 2 years continues.

All pregnancies were managed according to the local fetal medicine unit protocols. This included ultrasound assessment of biometry every 2 weeks and Doppler velocimetry every week, increasing to Doppler velocimetry twice a week or more with absent or reversed UmA EDF. Preeclampsia was defined according to International Society for the Study of Hypertension in Pregnancy (ISSHP) criteria (64), meaning that, given the presence of FGR, any woman developing new-onset hypertension after 20+0 weeks of gestation was classified as having preeclampsia rather than pregnancy-induced hypertension. Formalin-fixed placental samples were classified according to Amsterdam consensus criteria by a single assessor (65). To minimize bias, study placental samples were mixed with placental samples from healthy term pregnancies and pregnant women who delivered spontaneously preterm, with the investigator blinded to pregnancy phenotype and outcome during the assessment.

### Ultrasound measurements.

All ultrasound examinations were performed by staff trained and validated in the common EVERREST Prospective Study protocol (26). At each ultrasound scan, Doppler velocimetry of the UmA, UtA, middle cerebral artery (MCA), DV, and umbilical vein was performed (66). Local EFW formulas and centile charts were used to determine study eligibility, but for consistency, all EFWs were recalculated using the Hadlock 3 formula (incorporating head circumference, abdominal circumference, and femur length), with *z* scores recalculated using the Marsal chart for descriptive data (Supplemental Equations 1–3) (67, 68). EFWs and *z* scores were also recalculated using Intergrowth formulas for analysis (Supplemental Equations 4 and 5) (69). The effect of alternative Doppler reference charts was explored, with results similar to those presented previously (70–74).

### Demographic data.

Maternal ethnicity was self-reported according to the UK 2021 census list of ethnic groups, with the following options: White; Asian, including Indian, Pakistani, Bangladeshi, Chinese and any other Asian background; Black, including Caribbean, African, and any other Black background; multiethnic; and other (75).

### Blinding.

Maternal serum protein concentrations were not available to clinicians, participants, or researchers during the pregnancy, as all samples were analyzed after complete primary outcome data had been ascertained. Serum PlGF and sFLT1 concentrations were not used as part of clinical care at any of the study centers during the recruitment period.

### Sample collection.

Maternal blood was collected at study enrollment in BD Vacutainer serum-separating tubes and processed according to the manufacturer’s instructions. Serum aliquots (500 μL) were frozen and stored at –80°C. Placental samples for Amsterdam criteria categorization were collected from 2 areas of each placenta, midway between the cord insertion and margin in areas free from macroscopic infarcts or lesions. Samples were rinsed in PBS, formalin fixed, wax embedded, sectioned, and stained with H&E.

### Measurement of a priori candidate biomarkers in maternal serum.

PlGF and sFLT1 concentrations were measured using Elecsys electrochemiluminescence immunoassays on a Cobas e411 analyzer (Roche Diagnostics). The NPX of 90 additional proteins associated with cardiovascular disease was measured using the Olink Cardiovascular II proximity extension assay (full list of proteins in Supplemental Table 17). In the discovery set, but not the validation set, VEGFA, VEGFD, VEGFR2, neuropilin 1 (NRP1), and endoglin were measured in triplicate using Quantikine colorimetric sandwich ELISAs (R&D Systems).

### Identification of novel candidate biomarkers in maternal serum using liquid chromatography and tandem mass spectrometry.

Five pooled serum samples were created for the following pregnancy outcomes: (a) pregnancies ending in fetal or neonatal death; (b) pregnancies ending in neonatal survival with delivery before 37+0 weeks of gestation; (c) pregnancies ending in neonatal survival with delivery at 37+0 weeks of gestation or later; (d) slow fetal growth trajectory; and (e) normal fetal growth trajectory. Pooled serum samples were depleted of 12 high-abundance proteins using Proteome Purify 12 resin (R&S Systems), according to the manufacturer’s instructions, concentrated using Vivaspin 500 5 kDa Molecular Weight Cut-Off columns (GE Healthcare), reduced with 10 mM tris(2-carboxyethyl)phosphine hydrochloride, and then alkylated with 7.5 mM iodoacetamide. Pooled samples were digested using a trypsin/Lys-C mix, labeled with Tandem Mass Tags (Thermo Fisher Scientific), and combined (76). The combined sample underwent 2D high-performance reversed-phase liquid chromatography and tandem mass spectrometry. In the first dimension, samples were fractionated into 30 parts at high pH using a Poroshell 300 Extend C18 column (Agilent Technologies), following which fractions 1 to 4 were combined with fractions 27 to 30, respectively, given the low abundance in the first 4 fractions. The second fractionation was performed on the Ultimate 3000 nano-liquid chromatography system using Acclaim PepMap 100 C18 precolumns and Acclaim PepMap 100 C18 Nano-LC columns run in tandem with analysis on the linear trap quadrupole (LTQ) Orbitrap XL 2.5.5 (all from Thermo Fisher Scientific). A blank calibration sample was run after every 3 fractions, and a standard sample of known mass was run after every 6 fractions for quality control.

Proteins were identified using Proteome Discover version 1.4 software (Thermo Fisher Scientific) to search the human Swiss-Prot database with the Mascot search engine (Matrix Science). Proteins were scored on variability, peptide count, ubiquity, ratio between pools, and consistent trend across pools (Supplemental Tables 18 and 19). Expression pattern clusters, based on standardized and raw quantification ratios, were generated using the Graphical Proteomics Data Explorer (GPRoX) platform. On the basis of their scores and expression clusters, 5 candidate proteins were selected and measured in individual samples using ELISAs. Fibronectin, PSG1 (both from R&D Systems), and CSH (DRG International, measuring CSH1 and CSH2) were measured in the discovery and validation sets, while SAA (R&D Systems) and LNPEP (Cloud-Clone) were measured in the discovery set only. See Supplemental Table 20 for a summary of proteins analyzed for each study component.

### Priority survey and model selection.

An online survey was sent to patients and clinicians asking their opinion on the importance of different pregnancy outcomes and, for each outcome, whether they would prioritize sensitivity or specificity (see Supplemental Table 21 for full wording of the questions). Models were selected on the basis of the survey results and the model performance metrics described below. Protein models were published online prior to the validation data analysis.

### Sample size.

Since this work involved the discovery of novel biomarkers, a formal a priori sample size calculation was not possible. Before analyzing the discovery set, it was determined that this sample of 63 with 21 fetal or neonatal deaths gave an 80% power to detect a standardized effect size of 0.9 (large) to a significance level of 0.05 (77).

### Model development.

Two-protein models for the development of abnormal UmA Dopplers and 2- and 3-protein models for the other 3 pregnancy outcomes, with internal validation using LOOCV, were compared on the basis of AUC, specificity for 90% sensitivity, sensitivity for 90% specificity, F1 score, Matthews correlation coefficient (MCC), and precision-recall characteristics (PRROC) AUC. ROC curves were generated with the *pROC* R package (version 1.18.0, https://cran.r-project.org/web/packages/pROC/index.html). Ninety-five percent CIs for AUCs were determined by stratified bootstrapping. PRROC curves were generated with the MLmetrics R package (version 1.1.1, https://cran.r-project.org/web/packages/MLmetrics/index.html). Models with variance inflation factors of 5 or more were excluded using the following R package: https://cran.r-project.org/web/packages/car/index.html, version 3.1-0. Two-variable models predicting fetal or neonatal death and death or delivery at or before 28+0 weeks’ gestation containing ultrasound parameters with or without PlGF or CSH (as the proteins showing the strongest associations with these outcomes) were compared in the same way. Outcomes and protein models to be validated were published on the study registry prior to analysis of the validation data.

### Parenclitic network analysis.

Parenclitic networks of the 102 proteins were generated for each of the 4 pregnancy outcomes. For each outcome, 2D kernel density estimations were generated for every pair-combination of variables in “controls” (pregnancies without the outcome). Individual networks were then generated for each “case,” with linkages created if a pair-wise relationship of variables differed from the control distribution by more than a given threshold (80). These individual case networks were then combined. VEGFA, BNP, PARP1, and melusin were included as binary variables of “detectable” or “not detectable.” Booking BMI and fetal sex were included as variables in the networks, except for “development of abnormal UmA Dopplers,” where the sample was not large enough to accommodate them.

### Model validation.

Concentrations of CSH and PSG1, as measured by ELISA, and NPX values for the Olink multiplex proteins showed substantial variation in centrality and spread between the discovery and validation sets. To account for this, values of each protein were centered to a mean of 0 and scaled to a SD of 1 in the discovery set and validation set separately. These centered and scaled values were used for subsequent analyses, including model validation. Concentrations of PlGF, sFLT1, and fibronectin did not require transformation.

Models generated from the discovery set were run on data from the validation set and were considered validated if the 95% CI for the validation estimate of the AUC included the LOOCV AUC estimate from the discovery set. For validated models, data from both sets were combined to give final test characteristics. LR tests were used to determine whether the addition of pregnancy characteristics (maternal BMI, maternal age, maternal ethnicity, fetal sex, gestational age at enrollment, and preeclampsia at enrollment) significantly improved the validated models. Model calibration was assessed by plotting the predicted probability against the observed frequency of outcome.

### Functional interactions.

Centered and scaled data from the discovery and validation sets were combined to retest univariate associations with the primary and secondary outcomes. Proteins showing a significant association at a 5% Benjamini-Hochberg FDR were explored for physical and functional interactions and for enrichment of GO biological processes, relative to the background of all proteins measured, using STRING (Swiss Institute of Bioinformatics) (81). Where no enrichment was detected, shared GO biological processes were identified through comparison with the whole genome.

### Modeling pregnancy duration.

Protein and ultrasound measurements from the combined discovery and validation sets were tested for their association with gestational age at live birth or diagnosis of fetal death and interval from enrollment to live birth or diagnosis of fetal death using linear regression. Variables showing a significant association at a 1% Benjamini-Hochberg FDR were used to create linear models predicting these outcomes in a stepwise fashion. Maternal age, BMI, ethnicity, gestational age at enrollment, preeclampsia at enrollment, and fetal sex were tested for model improvement. Model fit was tested by assessing variance inflation factors for multicollinearity, assessing the distribution of the residuals for heteroscedasticity and outliers, and looking for observations with high leverage. UmA and UtA Doppler velocimetry, PlGF concentration, CSH concentration and PAPPA NPX were tested for their associations with placental histological classification using logistic regression.

### Statistics.

Data analysis was performed using STATA/MP 16.1 software (StataCorp) unless otherwise specified. Descriptive and investigative variables were tested for skew and kurtosis (78, 79) and handled as symmetrical if there was no evidence of either. PlGF, sFLT1, endoglin, VEGFD, NRP1, CSH, SAA, and LNPEP were transformed to their natural logs, and multiplex data were analyzed as provided, on a log_2_ scale. Characteristics of the discovery and validation sets were compared using χ^2^ tests (categorical data), Fisher’s exact tests (binary data with sparse outcomes), 2-sided *t* tests (symmetrical continuous data), and Mann-Whitney *U* tests (skewed continuous data).

Missing data for BMI (*n* = 5) were imputed using chain equations. UmA PI at enrollment was systematically missing (*n* = 16), with most missing cases having absent or reversed EDF (*n* = 15). UmA Doppler velocimetry was therefore handled as an interval variable, “UmA PI category,” where 0 = UmA PI at or below the 95th centile, 1 = UmA above the 95th centile with positive EDF, 2 = absent EDF, and 3 = reversed EDF. Where UtA PI at enrollment was missing (*n* = 10), a mean UtA PI below or above the 95th centile could be inferred in 9 cases in which the mean UtA PI was consistently normal (*n* = 1) or abnormal (*n* = 8), respectively, at scans prior to and after enrollment and UtA PI values were imputed using multiple imputation. Associations between ultrasound measurements and both fetal or neonatal death and death or delivery at or before 28+0 weeks of gestation were analyzed using logistic regression. Univariate associations between protein concentrations or NPX and outcomes were assessed using 2-sided *t* tests, Mann-Whitney *U* tests, and logistic regression, with Benjamini-Hochberg procedures to account for multiple comparisons.

### Study approval.

Ethics approval was provided by the National Research Ethics Service Committee London – Stanmore in the UK (REC reference: 13/LO/1254); the Hospital Clinic of Barcelona’s Clinical Research Ethics Committee in Spain (Reg: HCB/2014/0091); the Regional Ethical Review Board in Lund in Sweden (DNr 2014/147); and the Ethics Committee of the Hamburg Board of Physicians in Germany (PV4809). This study was conducted according to Declaration of Helsinki principles, and written informed consent was given by all participants before enrollment.

### Data availability.

The full data set will not be made publicly available because the degree of detailed phenotyping could allow individual patient identification. Limited data sharing may be possible, with the agreement of the EVERREST Consortium, upon transfer agreement request, directed to the corresponding author. Values for all data points in the figures can be found in the Supplemental Supporting Data Values file.

## Author contributions

RS contributed to study design, patient recruitment, data collection, sample analysis, data analysis, and writing of the manuscript. K Maksym contributed to patient recruitment and data collection. KH, K Maršál, FF, SRH, AD, JB, EG, DMP, IZ, and ALD contributed to study design, patient recruitment, and data collection. GA contributed to study design and data analysis. HW contributed to sample analysis and data analysis. NRN contributed to data analysis. NJS contributed to study design and sample analysis. YG and TW contributed to patient recruitment and data collection. NM and AHC contributed to study design. All authors critically revised the manuscript, gave approval for publication of the work, and agreed to be accountable for all aspects of it.

## Supplementary Material

Supplemental data

Trial reporting checklists

ICMJE disclosure forms

Supporting data values

## Figures and Tables

**Table 3:**
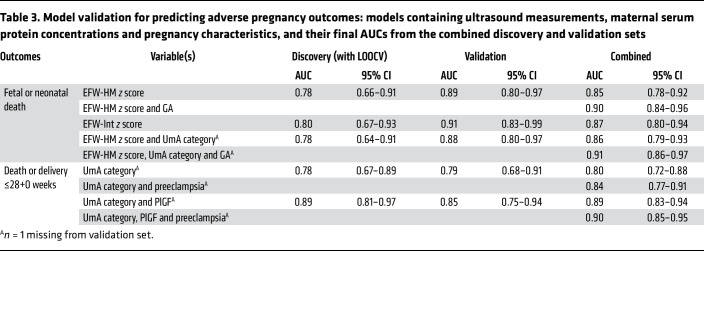
Model validation for predicting adverse pregnancy outcomes: models containing ultrasound measurements, maternal serum protein concentrations and pregnancy characteristics, and their final AUCs from the combined discovery and validation sets

**Table 2:**
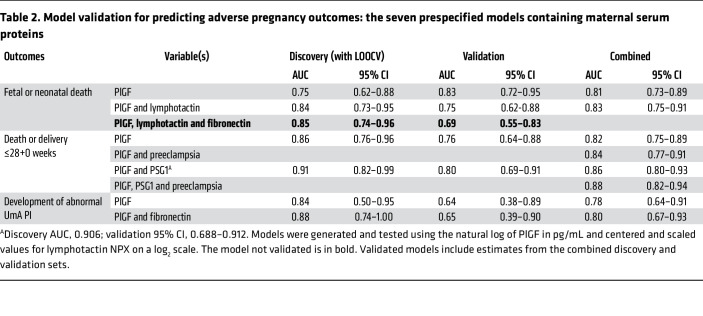
Model validation for predicting adverse pregnancy outcomes: the seven prespecified models containing maternal serum proteins

**Table 1:**
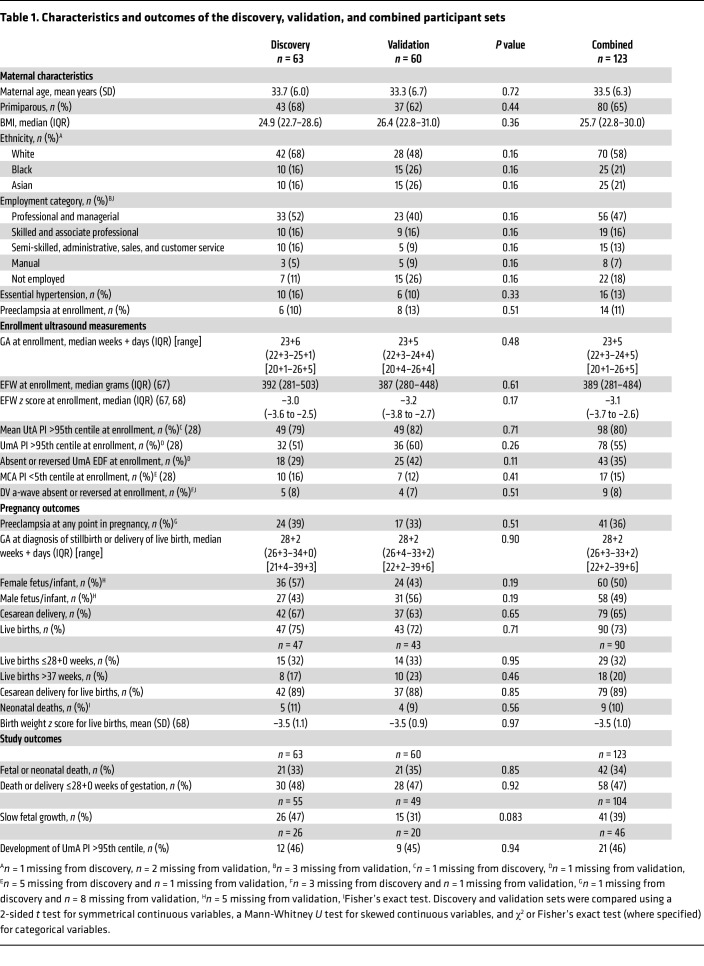
Characteristics and outcomes of the discovery, validation, and combined participant sets

## Version 1 09/15/2023

Electronic publication
