# Supplementary material for: Maternal PlGF and umbilical Dopplers predict pregnancy outcomes at diagnosis of early-onset fetal growth restriction
Source: J Clin Invest. 2023 Sep 15;133(18):e169199. doi: 10.1172/JCI169199 (PMC10503803; doi:10.1172/JCI169199)
Supplement: Supplemental data [file jci-133-169199-s123.pdf]

Supplementary data

Contents of Supplementary Data

Results

Table 1: Recruitment by study centre for the discovery and validation sets.......................................4

Table 2: Core maternal and neonatal outcomes recommended for reporting by the COSGROVE (Core Outcome Set for the prevention and treatment of fetal GROwth restriction: deVeloping Endpoints) consensus process..4

Figure 1: Gestational age range from enrolment to either live birth or diagnosis of intrauterine fetal death for all participants, sorted by gestational age at enrolment. 5

Table 3: Associations between ultrasound measurements at enrolment and fetal or neonatal death and death or delivery ≤28+0 weeks of gestation in the discovery set..6

Table 4: Associations between ultrasound measurements at enrolment and (1) fetal or neonatal death and (2) death or delivery ≤28+0 weeks of gestation in the combined discovery and validation sets. 7

Table 5: Abbreviations and full names of proteins from main article figures.......................................8

Figure 2: Dendrogram to accompany network analysis for pregnancies ending in fetal or neonatal death versus livebirths surviving to 29 days of life, based on edge betweenness analysis......... 10

Figure 3: Dendrogram to accompany network analysis for pregnancies ending in fetal death or delivery ≤28+0 weeks of gestation versus continuation of pregnancy beyond 28+0 weeks, based on edge betweenness analysis. 11

Figure 4: Network analysis for pregnancies that developed an abnormal UmA pulsatility index >95th centile versus those that did not. (A) Parenclitic protein network and clustering (B) Dendrogram, based on edge betweenness analysis. 12

Figure 5: Network analysis for pregnancies with slow fetal growth versus those without slow fetal growth. (A) Parenclitic protein network and clustering (B) Dendrogram, based on edge betweenness analysis. Slow fetal growth was defined as a worsening of weight deviation of >10 percentage points over a two-week interval or equivalent trajectory over a longer period (Marsal, 2009).. 13

Table 6: Experience and geographical origin of the 45 clinicians completing the marker priority survey.. 15

Figure 6: (A) The perceived importance of predicting term livebirth to patients, clinicians for the purpose of pregnancy management and clinicians for the purpose of patient counselling. (B) the proportion of respondents who prioritised either sensitivity or specificity for each pregnancy outcome.. 15

Table 7: Models significantly improved by the addition of gestational age or pre-eclampsia at enrolment. Likelihood ratio (LR) tests, Akaike information criteria (AIC) and Schwarz's Baysian information criteria (BIC) showing the effects of the added variables. 16

Table 8: Details of the validated models to predict fetal or neonatal death. Constants and coefficients for calculating log(odds) provided, along with variable cut points that give the maximum positive likelihood ratio (LR+), maximum correct classification and minimum negative likelihood ratio (LR-). 17

Table 9: Details of the validated models to predict death or delivery ≤28+0 weeks of gestation. Constants and coefficients for calculating log(odds) and variable cut points that give the maximum positive likelihood ratio (LR+), maximum correct classification and minimum negative likelihood ratio (LR-). 18

Table 10: Details of the validated models to predict development of umbilical artery pulsatility index >95th centile. Constants and coefficients for calculating log(odds) provided, along with variable

cut points that give the maximum positive likelihood ratio (LR+), maximum correct classification and minimum negative likelihood ratio (LR-). 19
Figure 7: Calibration plots for the models in Tables 8 and 9. 20
Figure 8: The differences between estimated fetal weights (EFWs) generated using the Intergrowth formula (Stirnemann et al., 2017) and the Hadlock 3 formula (Hadlock et al., 1985) and birthweight (BW) for livebirths with an EFW performed within seven days of delivery. (A) Absolute differences for n=21 pregnancies with a BW <600g (B) percentage difference, as a percentage of BW, for n=21 pregnancies with a BW <600g (C) absolute differences for all n=67 pregnancies (D) percentage difference, as a percentage of BW, for all n=67 pregnancies. 24
Figure 9: Calibration plots showing (A) the effect of using the estimated fetal weight calculated with the Hadlock 3 formula and z-score from the Marsal reference chart in the model generated from the Intergrowth z-score, and (B) the effect of using the Intergrowth z-score in the model generated from the Hadlock 3 formula estimated fetal weight z-score from the Marsal chart. 25
Table 11: Proteins showing an association with pregnancy outcomes at a Benjamini Hochberg 5% false discovery rate and Benjamini Hochberg 1% false discovery rate in centered and scaled data from the combined discovery and validation sets. 26
Figure 10: Volcano plots showing the statistical significance and magnitude of associations between the centred and scaled concentrations of the 93 proteins from the discovery and validation sets and (A) the development of abnormal umbilical artery (UmA) Dopplers (pulsatility index >95th centile) (B) slow fetal growth (a worsening of weight deviation of $\geq$10 percentage points over a two-week interval). 27
Table 12: Top three three-variable and top two-variable models for predicting fetal or neonatal death, death or delivery ≤28+0 weeks of gestation and the development of abnormal umbilical artery (UmA) Dopplers from the combined data set. Leave-one-out cross-validated models generated using centred and scaled values and evaluated on the basis of geometric mean of receiver operating characteristic curve area under the curve (AUC), precision-recall ROC area under the curve (PRROC), Matthews correlation coefficient (MCC) and F_1 rankings, excluding models with variance inflation factors (VIFs) of five or more (see Methods). 28
Table 13: Protein and ultrasound measurements at enrolment showing a significant association with gestational age at livebirth or diagnosis of fetal death and/or interval between enrolment and livebirth or diagnosis of fetal death at a Benjamini-Hochberg 1% false discovery rate. 29
Figure 11: (A) Predicted versus actual gestational age of either livebirth or diagnosis of fetal death, based on the sparser model containing PlGF and sFLT1 concentration and umbilical artery Doppler category (B) Predicted versus actual interval from enrolment to either livebirth or diagnosis of fetal death, based on the sparser model containing PlGF and sFLT1 concentrations, umbilical artery Doppler category and gestational age at enrolment. 29
Table 14: Comparison of the maternal and pregnancy characteristics of participants with and without samples available for placental histological classification. 30
Table 15: Pregnancy outcome by Amsterdam consensus placental classification (Khong et al., 2016) for the 55 pregnancies with available placental histology samples. 30
Table 16: Associations between ultrasound measurements and maternal serum protein concentrations at enrolment and subsequent placental classification of (1) any placental pathology (2) maternal vascular malperfusion (MVM) according to the Amsterdam consensus criteria (Khong et al., 2016). 31
Equation 1: Hadlock 3 EFW formula (Hadlock et al., 1985) 32
Equation 2: Marsal EFW z-score (Marsal et al., 1996) 32
Equation 3: Percentage weight deviation (Marsal, 2009) 32
Equation 4: Intergrowth EFW formula (Stirnemann et al., 2017) 32
Equation 5: Intergrowth z-score formula (Stirnemann et al., 2017) 33
Table 17: Full list of proteins measured by the Olink Proseek® Multiplex cardiovascular disease (CVD) II proximity extension assay with reported intra-assay variation 33

Table 18: The scoring system for ranking proteins identified through pooled mass spectrometry 35
Table 19: Top 50 highest scoring proteins identified from pooled serum liquid chromatography and tandem mass spectrometry, based on the scoring system above. 36
Table 20: Combinations of proteins included in different sections of the study 38
Table 21: Marker priority survey questions for clinicians and patients 39

Results

Table 1: Recruitment by study centre for the discovery and validation sets.

Centre	Discovery (n %)	Validation (n %)	Combined (n %)
University College London Hospital, UK	48 (76)	52 (87)	100 (81)
Maternal-Fetal Unit Hospital Clinic Barcelona, Spain	6 (10)	4 (7)	10 (8)
University Medical Center Hamburg-Eppendorf, Germany	5 (8)	2 (3)	7 (6)
Skane University Hospital, Lund, Sweden	4 (6)	2 (3)	6 (5)

Table 2: Core maternal and neonatal outcomes recommended for reporting by the COSGROVE (Core Outcome Set for the prevention and treatment of fetal GROwth restriction: deVeloping Endpoints) consensus process. Childhood outcomes at 2 years of age are still being assessed and will be reported in due course.

Domain	Outcome	
Maternal (n=123)	Eclampsia (n)	0
	Maternal death (n)	0
Neonatal (n=90)	Birthweight (g, median IQR)	953 (590-1650)
	Birthweight <3rd centile (n %)	83 (92%)
	Birthweight <10th centile (n %)	88 (98%)
	Need for mechanical ventilation (n %)	66 (73%)
	Bronchopulmonary dysplasia / chronic lung disease (n%)*	47 (59%)
	Necrotising enterocolitis (n %)	10 (11%)

*n=9 neonatal deaths before 36 weeks corrected age, n=2 missing.

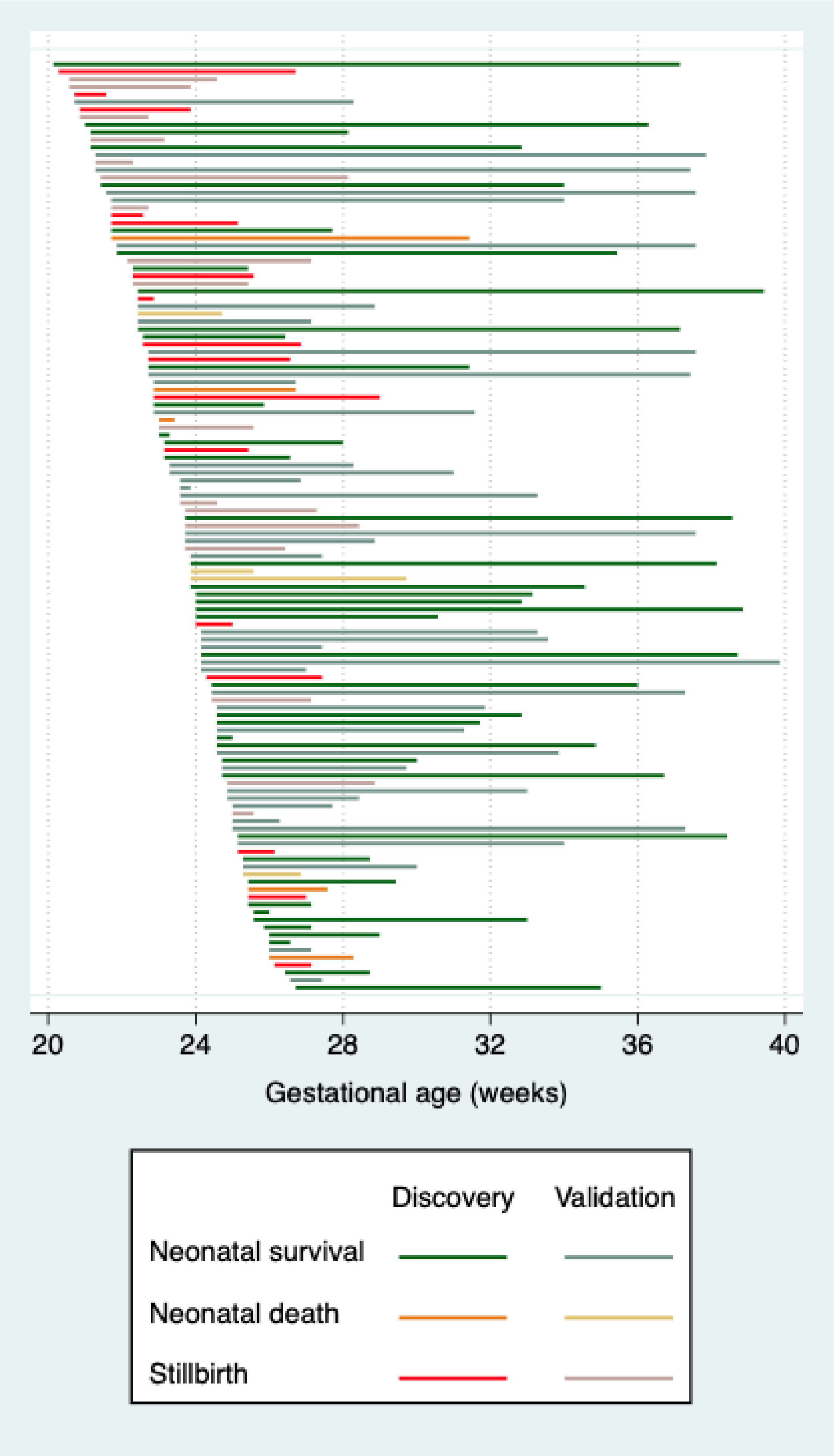

Figure 1: Gestational age range from enrolment to either live birth or diagnosis of intrauterine fetal death for all participants, sorted by gestational age at enrolment.

Table 3: Associations between ultrasound measurements at enrolment and fetal or neonatal death and death or delivery ≤28+0 weeks of gestation in the discovery set.

Ultrasound variables		Fetal or neonatal death				Death or delivery ≤28+0 weeks of gestation			
		OR (95% CI)	p value	RR (95% CI)	AUC (95% CI)	OR (95% CI)	p value	RR (95% CI)	AUC (95% CI)
UmA PI >95th centile (Schaffer and Staudach, 1997)	n=63	5.2 (1.6-17.0)	0.006	3.1 (1.4-8.5)	0.69 (0.57-0.81)	8.8 (2.8-27.4)	0.0002	3.2 (1.4-7.4)	0.75 (0.64-0.86)
Absent or reversed UmA EDF	n=63	5.5 (1.7-17.9)	0.005	2.8 (1.7-6.5)	0.68 (0.55-0.80)	17.7 (3.6-87.7)	0.0004	2.9 (1.4-5.9)	0.74 (0.64-0.84)
UmA category[1]	n=63	2.8 (1.6-5.2)	0.001	1.8 (1.2-2.6)	0.75 (0.62-0.88)	4.4 (2.1-9.3)	0.0001	1.7 (1.2-2.3)	0.80 (0.70-0.91)
MCA PI <5th centile (Schaffer and Staudach, 1997)	n=58	1.3 (0.3-5.4)	0.69	1.2 (0.4-3.6)		2.8 (0.64-12.0)	0.18	1.5 (0.7-3.6)	
Absent or reversed DV a wave	n=60	10.7 (1.1-103.3)	0.04	2.9 (1.0-8.8)	0.59 (0.50-0.69)	4.8 (0.50-45.8)	0.18	1.8 (0.6-5.1)	
Mean UtA PI	n=62	1.7 (0.7-4.1)	0.23	1.4 (0.7-2.8)		6.4 (2.1-19.3)	0.001	2.1 (1.2-3.8)	0.77 (0.65-0.89)
Mean UtA PI >95th centile (Schaffer and Staudach, 1997)	n=62	1.8 (0.4-7.3)	0.43	1.5 (0.4-5.1)		1.4 (1.1-1.7)	0.002	7.4 (1.0-54.6)	0.76 (0.64-0.89)
EFW_{HM} z-score (Hadlock et al., 1985, Marsal et al., 1996)	n=63	0.17 (0.06-0.45)	0.0003	0.56 (0.40-0.77)	0.81 (0.69-0.93)	0.35 (0.17-0.72)	0.005	0.71 (0.52-0.95)	0.69 (0.56-0.82)
EFW_I z-score (Stirnemann et al., 2017)	n=63	0.37 (0.20-0.67)	0.001	0.80 (0.71-0.91)	0.83 (0.71-0.95)	0.54 (0.36-0.83)	0.004	0.87 (0.77-0.97)	0.73 (0.60-0.85)
Slow fetal growth[2]	n=55	5.4 (1.4-19.8)	0.012	3.3 (1.1-10.4)	0.70 (0.56-0.83)	2.6 (0.86-7.8)	0.09	1.7 (0.8-4.0)	

[1]Four levels: ≤95th centile, >95th centile with positive EDF, absent EDF, reversed EDF. [2]Slow fetal growth, defined as a worsening of percentage weight deviation by ≥10 percentage points over 2 weeks or equivalent (Marsal, 2009), was assessed over a minimum of 2 weeks so included data from follow-up scans. DV=ductus venosus, EDF=end-diastolic flow, EFW_{HM}=estimated fetal weight calculated using Hadlock 3 formula with z-score calculated using Marsal reference chart, EFW_I=estimated fetal weight and z-score calculated using Intergrowth formulae, MCA=middle cerebral artery, OR=odds ratio, PI=pulsatility index, RR=risk ratio (estimated using Poisson regression), UmA=umbilical artery, UtA=uterine artery.

Table 4: Associations between ultrasound measurements at enrolment and (1) fetal or neonatal death and (2) death or delivery ≤28+0 weeks of gestation in the combined discovery and validation sets. This is provided for reference but was not available at the time of model selection as models were selected prior to validation set recruitment.

Ultrasound variable		Fetal or neonatal death				Death or delivery ≤28+0 weeks of gestation			
		OR (95% CI)	p value	RR (95% CI)	AUC (95% CI)	OR (95% CI)	p value	RR (95% CI)	AUC (95% CI)
UmA PI $>95^{th}$ centile (Schaffer and Staudach, 1997)	n=122	6.7 (2.7-16.9)	0.0001	3.9 (1.7-8.7)	0.70 (0.63-0.78)	8.2 (3.5-18.8)	<0.00005	3.3 (1.7-6.4)	0.73 (0.66-0.81)
Absent or reversed UmA EDF	n=122	6.5 (2.8-15.0)	<0.00005	3.2 (1.7-6.0)	0.71 (0.63-0.80)	14.2 (5.5-36.8)	<0.00005	3.1 (1.8-5.4)	0.76 (0.69-0.84)
UmA category[1]	n=122	2.8 (1.1-4.2)	<0.00005	1.8 (1.4-2.4)	0.77 (0.69-0.86)	3.5 (2.2-5.6)	<0.00005	1.7 (1.3-2.1)	0.80 (0.72-0.88)
MCA PI $<5^{th}$ centile (Schaffer and Staudach, 1997)	n=117	1.8 (0.64-5.10)	0.27	1.4 (0.66-3.1)		2.2 (0.77-6.5)	0.14	1.4 (0.74-2.8)	
Absent or reversed DV a wave	n=119	19.5 (2.3-162.3)	0.006	3.1 (1.4-6.6)	0.59 (0.53-0.66)	4.4 (0.87-21.9)	0.07	1.7 (0.79-3.9)	
Mean UtA PI	n=122	2.3 (1.1-4.7)	0.017	1.7 (1.0-2.9)	0.64 (0.53-0.75)	4.1 (1.9-8.6)	0.0002	1.9 (1.2-3.0)	0.72 (0.62-0.81)
Mean UtA PI $>95^{th}$ centile (Schaffer and Staudach, 1997)	n=122	2.2 (0.76-6.4)	0.15	1.8 (0.69-4.5)		8.6 (2.4-30.7)	0.0009	4.4 (1.4-14.1)	0.64 (0.57-0.70)
EFW_{HM} z-score (Hadlock et al., 1985, Marsal et al., 1996)	n=123	0.11 (0.048-0.25)	<0.00005	0.50 (0.39-0.65)	0.85 (0.78-0.92)	0.32 (0.18-0.56)	<0.00005	0.67 (0.52-0.86)	0.64 (0.57-0.70)
EFW_{I} z-score (Stirnemann et al., 2017)	n=123	0.29 (0.18-0.47)	<0.00005	0.84 (0.79-0.90)	0.87 (0.80-0.94)	0.53 (0.39-0.71)	<0.00005	0.89 (0.83-0.96)	0.74 (0.66-0.83)
Slow fetal growth[2]	n=104	4.0 (1.68-9.74)	0.0018	2.6 (1.3-5.2)	0.67 (0.57-0.77)	2.3 (1.0-5.1)	0.049	1.6 (0.87-3.0)	0.60 (0.50-0.70)

[1]Four levels: ≤95th centile, >95th centile with positive EDF, absent EDF, reversed EDF. [2]Slow fetal growth, defined as a worsening of percentage weight deviation by ≥10 percentage points over 2 weeks or equivalent (Marsal, 2009), was assessed over a minimum of 2 weeks so included data from follow-up scans. DV=ductus venosus, EDF=end-diastolic flow, EFW_{HM}=estimated fetal weight calculated using Hadlock 3 formula with z-score calculated using Marsal reference chart, EFW_{I}=estimated fetal weight and z-score calculated using Intergrowth formulae, MCA=middle cerebral artery, OR=odds ratio, PI=pulsatility index, RR=risk ratio (estimated using Poisson regression), UmA=umbilical artery, UtA=uterine artery.

Table 5: Abbreviations and full names of proteins from main article figures.

Abbreviation	Full protein name	Figure				
		2	3	7	8	9
ACE2	Angiotensin-converting enzyme 2		x			
ADAMTS13	A disintegrin and metalloproteinase with thrombospondin motifs 13		x			
ADM	Pro-adrenomedullin		x	x		
AGRP	Agouti-related protein		x			
AMBP	Protein AMBP					x
ANGPT1	Angiopoietin 1		x			
BOC	Brother of cysteine dioxygenase	x	x			
CA5A	Carbonic anhydrase 5A, mitochondrial		x			x
CALCA	Calcitonin				x	
CALCRL	Calcitonin gene-related peptide type 1 receptor				x	
CD40L	CD40 ligand		x			x
CD47	Leucocyte surface antigen CD47				x	
CEACAM8	Carcinoembryonic antigen related cell adhesion molecule 8		x			
CHD5	Chromodomain-helicase-DNA-binding protein 5				x	
CSH / HPL	Chorionic somatomammotropin hormone / human placental lactogen	x	x	x	x	x
CTRC	Chymotrypsin C		x			
CTSL1	Cathepsin L1		x			
DCN	Decorin		x			x
DECR1	2,4-dienoyl-CoA reductase, mitochondrial		x			
DKK1	Dickkopf-related protein 1		x			
FABP2	Fatty acid-binding protein, intestinal	x	x			
FGF21	Fibroblast growth factor 21	x	x			x
FGF23	Fibroblast growth factor 23		x			
FN	Fibronectin					x
FST	Follistatin	x				x
GDF2	Growth/differentiation factor 2		x			x
GH1	Growth hormone		x	x		x
GHR	Growth hormone receptor				x	
GT / FABP6	Gastrotropin		x			
HAO1	Hydroxyacid oxidase 1			x		x
HAVCR1	Hepatitis A virus cellular receptor 1		x			
IKBKG	Inhibitor of nuclear factor kappa B kinase regulatory subunit G		x			
IL1RA	Interleukin 1 receptor antagonist protein	x				
IL1RL2	Interleukin 1 receptor-like 2	x	x			x
IL4RA	Interleukin 4 receptor subunit alpha					
IL6	Interleukin 6	x				
IL16	Pro-interleukin 16		x			
IL17D	Interleukin 17D		x	x		x
IL18	Interleukin 18		x			
IL27	Interleukin 27		x			
JAK2	Tyrosine-protein kinase JAK2				x	
LEP	Leptin		x			
LGALS9	Galectin 9	x				x
LNPEP	Leucyl-cystinyl aminopeptidase		x			
MARCO	Macrophage receptor with collagenous structure	x	x	x		
MMP12	Matrix metalloproteinase 12	x	x			x
NRP1	Neuropilin 1	x	x		x	
OLR1	Oxidized low density lipoprotein receptor 1	x	x			x
OSCAR	Osteoclast-associated immunoglobulin-like receptor		x			
PAPPA	Pappalysin-1 / pregnancy-associated plasma protein A	x	x			
PAR1	Proteinase-activated receptor 1		x			

PDGFB	Platelet-derived growth factor subunit B	x	x			
PDCD1LG2	Programmed cell death 1 ligand 2	x	x			x
PlGF	Placental growth factor	x	x	x		x
PLXNA1	Plexin-A1				x	
PRELP	Prolargin		x			
PRSS8	Serine protease 8		x			
PRSS27	Serine protease 27		x			
PSG1	Pregnancy-specific beta-glycoprotein 1	x		x		x
PTX3	Pentraxin-related protein PTX3		x			
RAGE	Receptor for advanced glycosylation end products		x			
RAMP1	Receptor activity-modifying protein 1				x	
RAMP2	Receptor activity-modifying protein 2				x	
RAMP3	Receptor activity-modifying protein 3				x	
REN	Renin		x			
SCF	Stem cell factor		x			
SEMA3A	Semaphorin 3A				x	
SERPINA12	Serpin A12		x			
sFLT1	Soluble fms-like tyrosine kinase 1				x	x
SH2D2A	SH2 domain-containing protein 2A				x	
SIRPG	Signal-regulatory protein gamma				x	
SOD2	Superoxide dismutase 2		x			
SPON2	Spondin 2		x			
SRC	Proto-oncogene tyrosine-protein kinase SRC		x			
STK4	Serine/threonine-protein kinase 4	x	x			
TF	Tissue factor		x			
TGM2	Protein-glutamine gamma-glutamyltransferase 2		x			
THBS1	Thrombospondin 1				x	
THBS2	Thrombospondin 2	x	x	x		x
TIE2	Angiopoietin 1 receptor		x			
TM	Thrombomodulin		x			
TNFRSF10A	Tumor necrosis factor receptor superfamily member 10A					x
TNFRSF10B	Tumor necrosis factor receptor superfamily member 10B		x			x
TNFRSF11A	Tumor necrosis factor receptor superfamily member 11A		x			
VEGFB	Vascular endothelial growth factor B				x	
VEGFD	Vascular endothelial growth factor D				x	
VEGFR2	Vascular endothelial growth factor receptor 2	x	x		x	
VSIG2	V-set and immunoglobulin domain-containing protein 2		x			
XCL1	Lymphotactin	x				

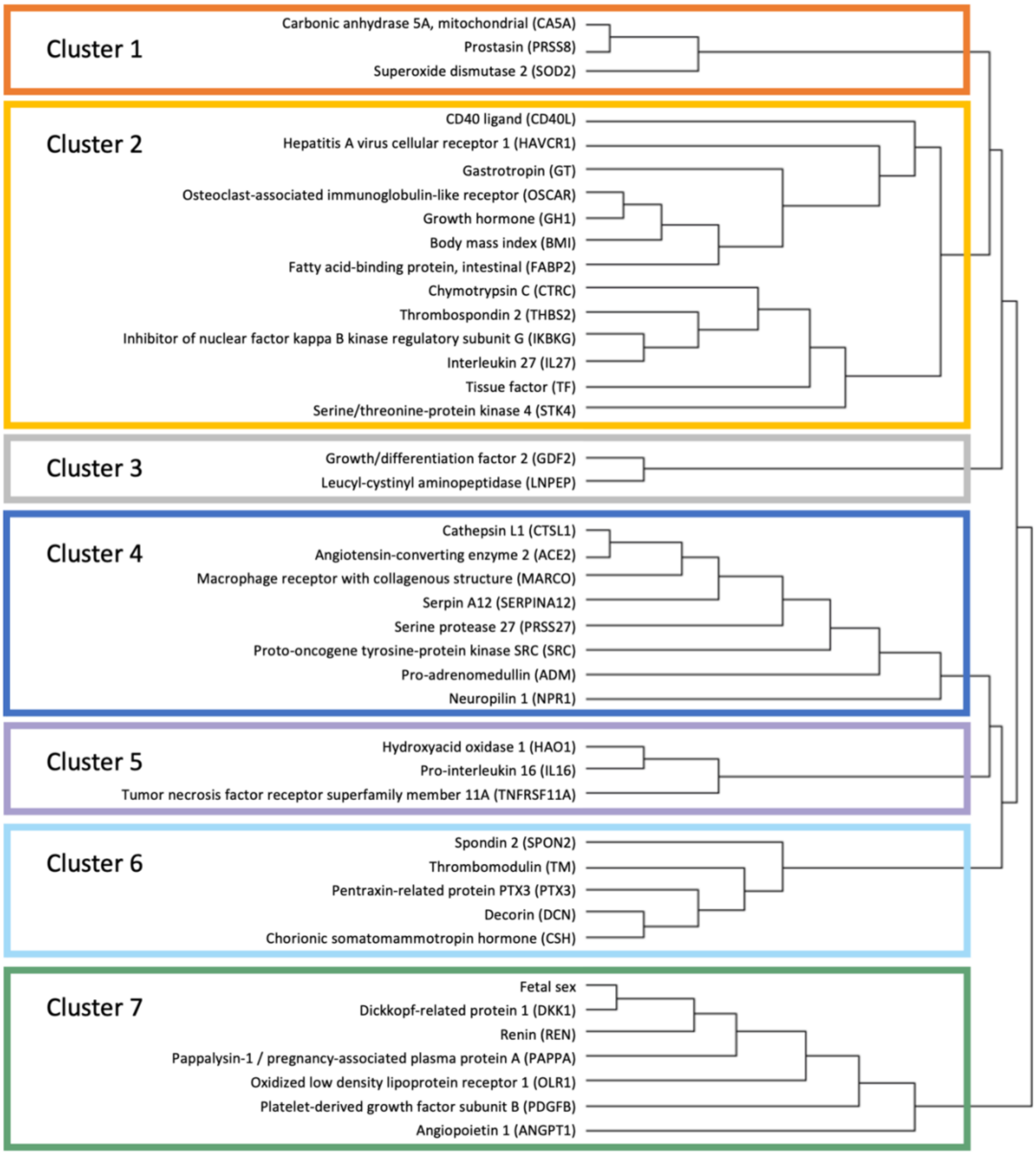

Figure 2: Dendrogram to accompany network analysis for pregnancies ending in fetal or neonatal death versus livebirths surviving to 29 days of life, based on edge betweenness analysis.

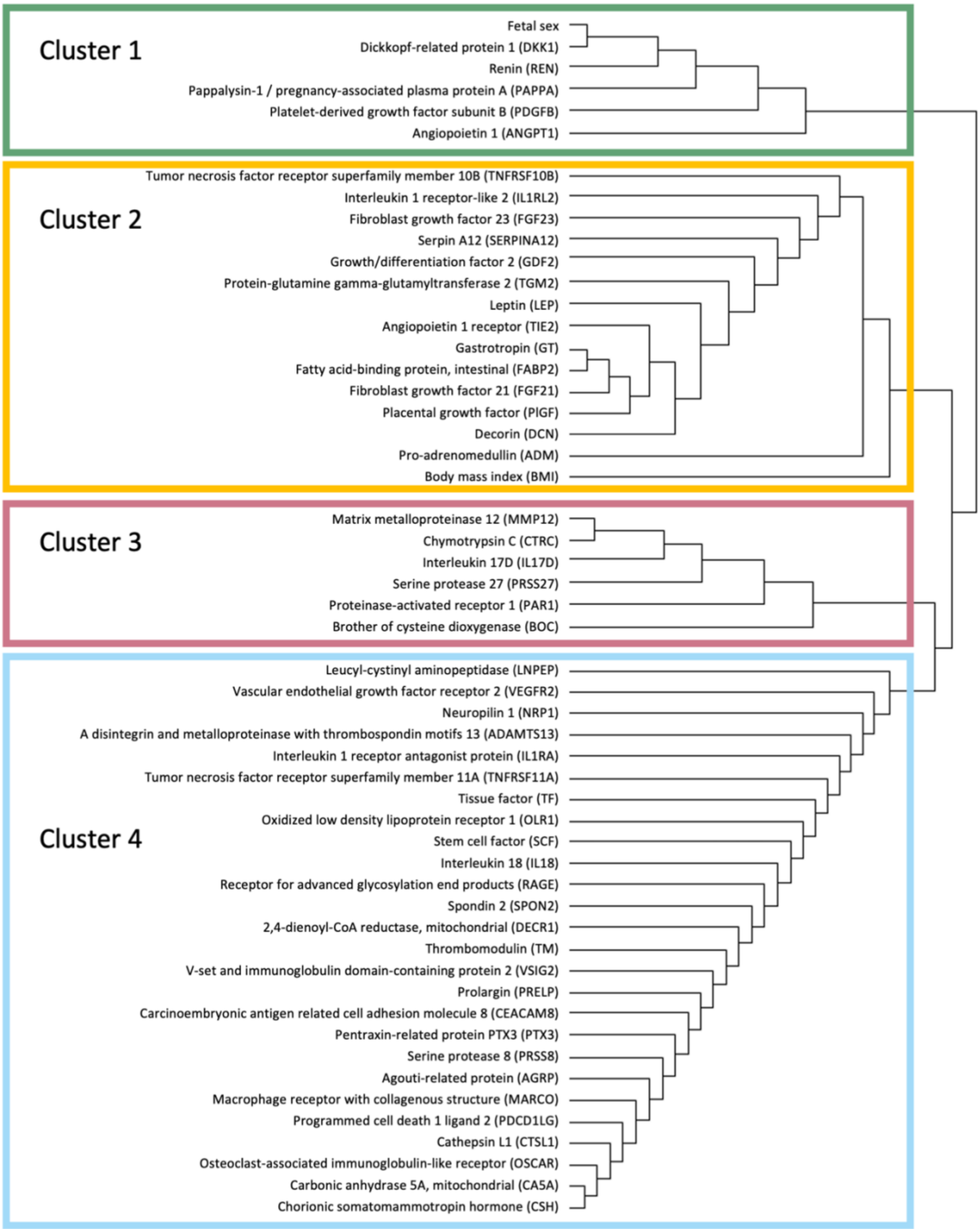

Figure 3: Dendrogram to accompany network analysis for pregnancies ending in fetal death or delivery ≤28+0 weeks of gestation versus continuation of pregnancy beyond 28+0 weeks, based on edge betweenness analysis.

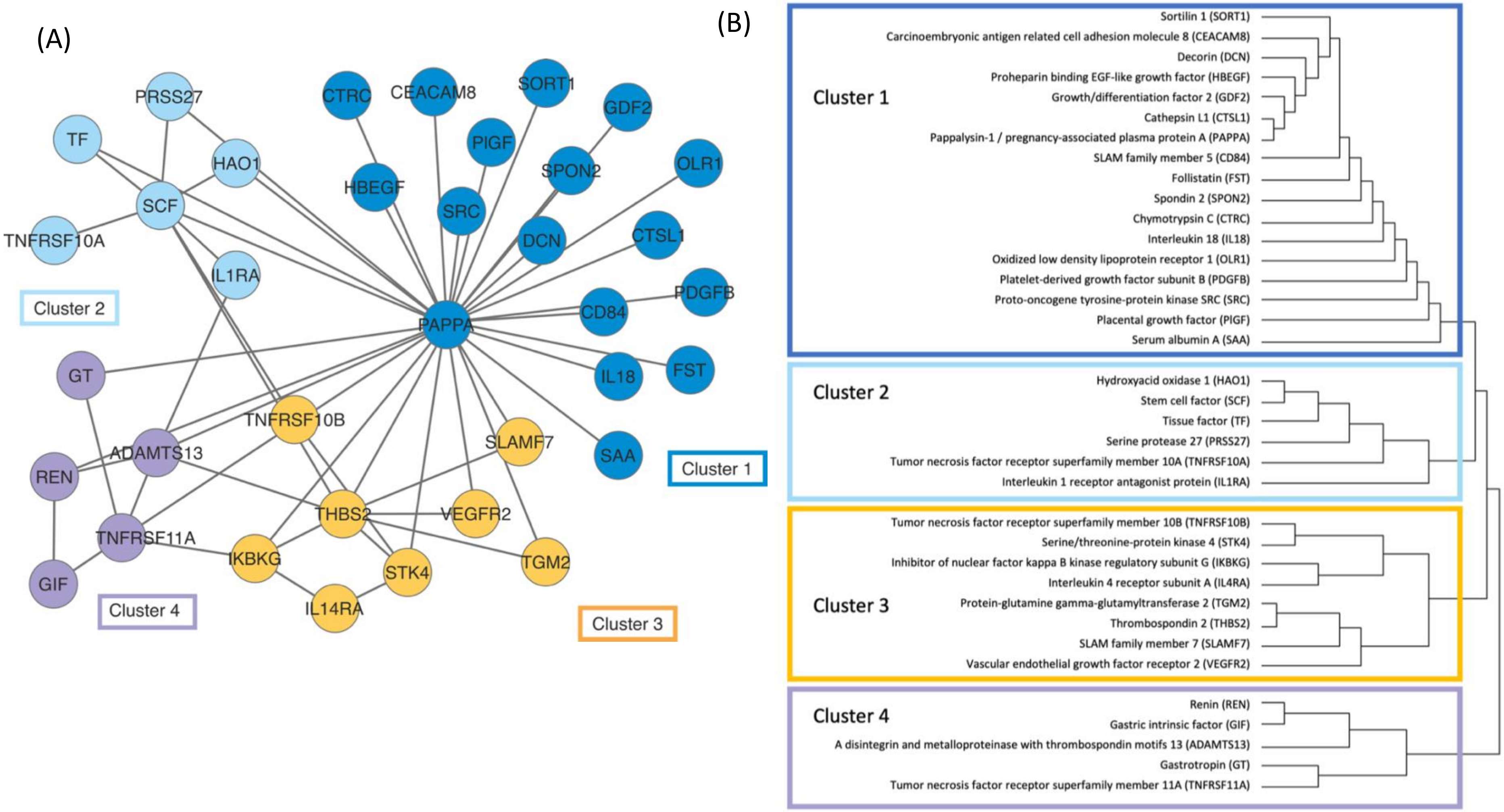

Figure 4: Network analysis for pregnancies that developed an abnormal UmA pulsatility index >95th centile versus those that did not. (A) Parenclitic protein network and clustering (B) Dendrogram, based on edge betweenness analysis.

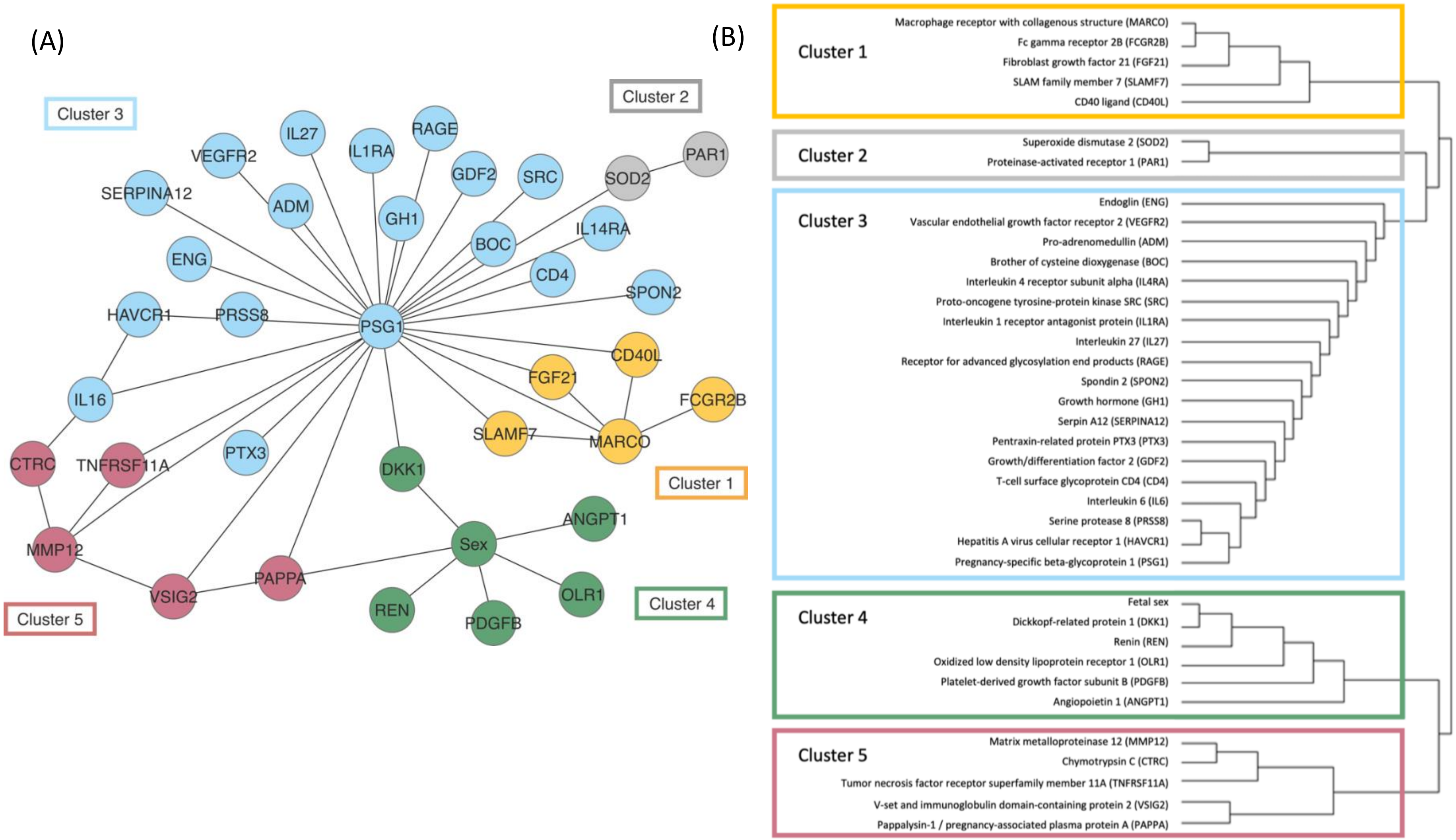

Figure 5: Network analysis for pregnancies with slow fetal growth versus those without slow fetal growth. (A) Parenclitic protein network and clustering (B) Dendrogram, based on edge betweenness analysis. Slow fetal growth was defined as a worsening of weight deviation of >10 percentage points over a two-week interval or equivalent trajectory over a longer period (Marsal, 2009).

Table 6: Experience and geographical origin of the 45 clinicians completing the marker priority survey.

Specialty	
	n (%)
Consultant in Maternal and Fetal Medicine	31 (69)
Consultant in Obstetrics and Gynaecology	8 (18)
Other	6 (13)

Location	
	n (%)
Europe	21 (47)
Australia and New Zealand	6 (13)
Asia and Middle East	5 (11)
North and South America	4 (36)

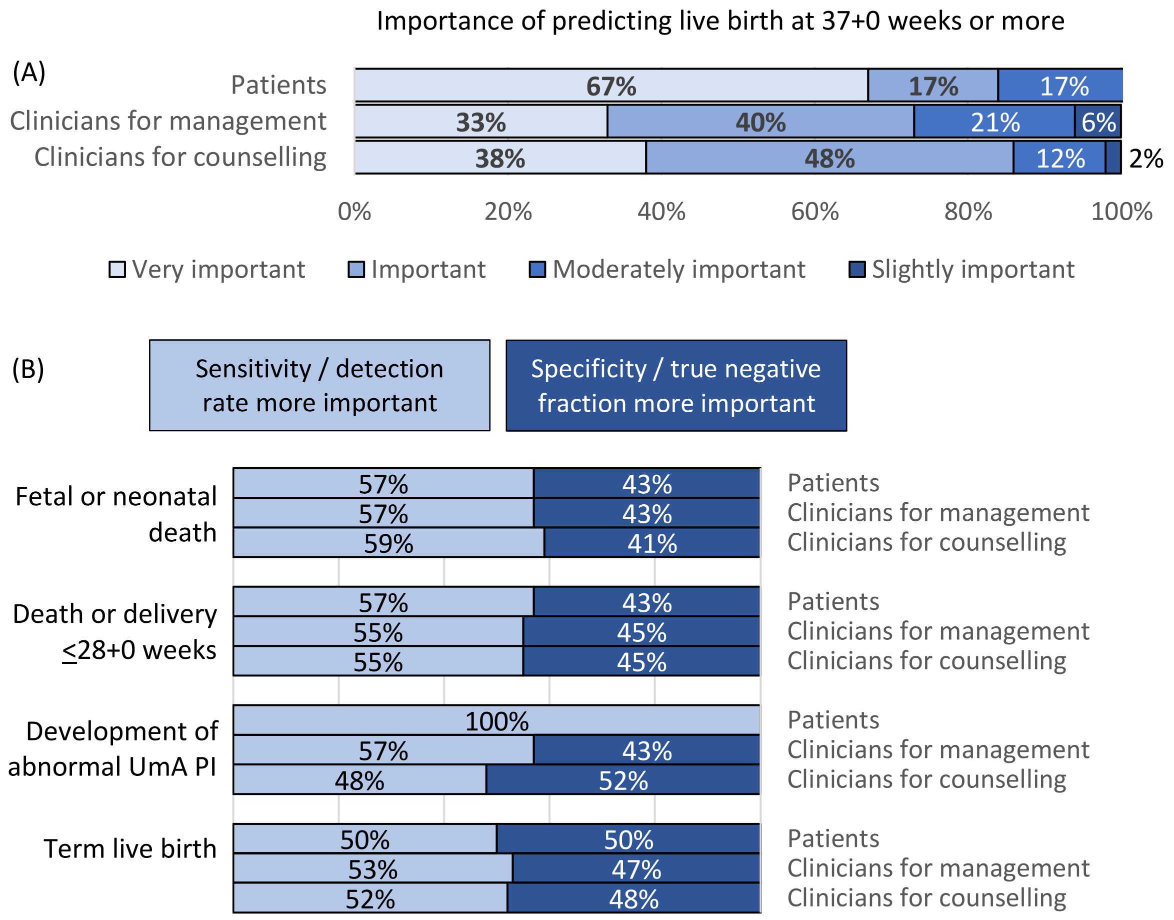

Figure 6: (A) The perceived importance of predicting term livebirth to patients, clinicians for the purpose of pregnancy management and clinicians for the purpose of patient counselling. (B) the proportion of respondents who prioritised either sensitivity or specificity for each pregnancy outcome. UmA PI=umbilical artery pulsatility index.

Table 7: Models significantly improved by the addition of gestational age or pre-eclampsia at enrolment. Likelihood ratio (LR) tests, Akaike information criteria (AIC) and Schwarz's Baysian information criteria (BIC) showing the effects of the added variables.

Outcome	Variable(s)	LR test p value	AIC	BIC
Fetal or neonatal death	EFW_{HM} z-score	0.0001	111.2	116.9
	EFW_{HM} z-score & gestational age		97.2	105.6
	EFW_{HM} z-score & UmA category[1]	<0.00005	107.7	116.1
	EFW_{HM} z-score & UmA category & gestational age[1]		90.7	101.9
Death or delivery ≤28+0 weeks of gestation	PlGF	0.013	128.9	134.5
	PlGF & pre-eclampsia		124.7	133.1
	PlGF & PSG1*	0.0042	119.3	127.7
	PlGF & PSG1 & pre-eclampsia		113.1	124.3
	UmA category[1]	0.0002	131.5	137.1
	UmA category & pre-eclampsia[1]		119.5	127.9
	UmA category & PlGF[1]	0.013	107.4	115.8
	UmA category & PlGF & pre-eclampsia[1]		103.2	114.4

[1]n=1 missing from validation set. EFW_{HM}=estimated fetal weight calculated using Hadlock 3 formula (Hadlock et al., 1985) with z-score calculated using Marsal reference chart (Marsal et al., 1996), EFW_{Int}=estimated fetal weight and z-score calculated using Intergrowth formulae (Stirnemann et al., 2017), PlGF=placental growth factor, PSG1=pregnancy-specific glycoprotein 1, UmA=umbilical artery.

Table 8: Details of the validated models to predict fetal or neonatal death. Constants and coefficients for calculating log(odds) provided, along with variable cut points that give the maximum positive likelihood ratio (LR+), maximum correct classification and minimum negative likelihood ratio (LR-). Calibration plots are provided in Supplemental Figure 7.

Variable(s)	Constant (95% CI)	Coefficient(s) (95% CI)	Cut point	Value of the variable	Correctly classified	LR+	LR-	Sensitivity	Specificity
PlGF	4.20 (2.21 to 6.19)	ln(PlGF): -1.43 (-2.04 to -0.826)	Max LR+	<14.2 pg/ml	80%	18.3	0.56	45%	98%
			Max correct						
			Min LR-	<240 pg/ml	43%	1.2	0.00	100%	14%
PlGF & lymphotactin	4.09 (2.07 to 6.10)	ln(PlGF): -1.41 (-2.03 to -0.795) lymphotactin: 0.442 (-0.061 to 0.945)	Max LR+		70%	11.6	0.87	14%	99%
			Max correct		80%	7.4	0.49	55%	93%
			Min LR-		47%	1.2	0.00	100%	20%
EFW_{HM} z-score	-8.02 (-10.83 to -5.20)	EFW_{HM} z: -2.21 (-3.03 to -1.38)	Max LR+	<-4.10	78%	30.9	0.63	38%	99%
			Max correct	<-3.46	81%	5.6	0.35	69%	88%
			Min LR-	<-2.25	44%	1.2	0.00	100%	15%
EFW_{HM} z-score & gestational age at enrolment	5.36 (-2.05 to 12.78)	EFW_{HM} z: -2.69 (-3.70 to -1.68) GA: -0.915 (-0.141 to -0.042)	Max LR+		78%	30.9	0.63	38%	99%
			Max correct		85%	8.3	0.31	71%	91%
			Min LR-		47%	1.2	0.00	100%	20%
EFW_{Int} z-score	-5.72 (-7.73 to -3.72)	EFW_{Int} z: -1.24 (-1.71 to -0.76)	Max LR+	<-5.16	84%	44.4	0.46	55%	99%
			Max correct						
			Min LR-	<-1.68	9%	1.1	0.00	100%	9%
EFW_{HM} z-score & UmA category[1]	-7.30 (-10.16 to -4.45)	EFW_{HM} z: -1.79 (-2.67 to -0.910) UmA: 0.56 (0.083 to 1.04)	Max LR+		82%	20.7	0.50	51%	98%
			Max correct		84%	6.8	0.27	76%	89%
			Min LR-		42%	1.1	0.00	100%	12%
EFW_{HM} z-score & UmA category & gestational age at enrolment[1]	8.45 (0.264 to 16.63)	EFW_{HM} z: -2.27 (-3.34 to -1.21) UmA: 0.784 (0.229 to 1.34) GA: -0.108 (-0.164 to -0.520)	Max LR+		83%	41.5	0.49	51%	99%
			Max correct		85%	5.7	0.17	85%	85%
			Min LR-		48%	1.3	0.00	100%	21%

[1]n=1 missing from validation set. PlGF=placental growth factor, UmA=umbilical artery. Models generated using the natural log of PlGF in pg/ml but cut points converted back to concentration. Models generated using centered and scaled values of lymphotactin normalised protein expression.

Table 9: Details of the validated models to predict death or delivery ≤28+0 weeks of gestation. Constants and coefficients for calculating log(odds) and variable cut points that give the maximum positive likelihood ratio (LR+), maximum correct classification and minimum negative likelihood ratio (LR-). Calibration plots are provided in Supplemental Figure 7.

Variable(s)	Constant (95% CI)	Coefficient(s) (95% CI)	Cut point	Value of variable	Correctly classified	LR+	LR-	Sensitivity	Specificity
PlGF	5.17 (3.17 to 7.17)	ln(PlGF): -1.50 (-2.07 to -0.924)	Max LR+	<14.5 pg/ml	70%	24.7	0.63	38%	98%
			Max correct	<34 pg/ml	76%	3.2	0.30	78%	75%
			Min LR-	<240 pg/ml	56%	1.2	0.00	100%	17%
PlGF & pre-eclampsia	4.58 (2.57 to 6.58)	ln(PlGF): -1.38 (-1.95 to -0.81)<br>Pre-eclampsia: 2.13 (2.57 to 4.24)	Max LR+		72%	26.9	0.60	41%	98%
			Max correct		76%	3.1	0.32	76%	76%
			Min LR-		56%	1.2	0.00	100%	17%
PlGF & PSG1	4.47 (2.38 to 6.56)	ln(PlGF): -1.30 (-1.88 to -0.714)<br>PSG1: -0.846 (-1.37 to -0.318)	Max LR+		67%	21.3	0.68	33%	98%
			Max correct		84%	4.4	0.15	88%	80%
			Min LR-		53%	1.1	0.00	100%	11%
PlGF, PSG1 & pre-eclampsia	3.66 (1.55 to 5.78)	ln(PlGF): -1.13 (-1.71 to -0.55)<br>PSG1: -0.95 (-1.51 to -0.40)<br>Pre-eclampsia: 2.91 (0.30 to 5.53)	Max LR+		74%	30.3	0.54	47%	98%
			Max correct		83%	4.3	0.17	86%	80%
			Min LR-		53%	1.1	0.00	100%	11%
UmA category[1]	-1.42 (-2.03 to -0.81)	UmA: 1.26 (0.81 to 1.72)	Max LR+	UmA PI >95th centile	63%	8.0	0.78	25%	97%
			Max correct	Absent or reversed EDF	77%	5.9	0.41	63%	89%
			Min LR-	Reversed EDF	73%	2.4	0.29	81%	66%
UmA category & pre-eclampsia[1]	-1.76 (-2.45 to -1.06)	UmA: 1.32 (0.83 to 1.81)<br>Pre-eclampsia: 3.22 (1.02 to 5.43)	Max LR+		69%	12.0	0.65	37%	97%
			Max correct		81%	6.0	0.30	74%	88%
			Min LR-		75%	2.4	0.22	86%	65%
UmA category & PlGF[1]	3.49 (1.29 to 5.70)	UmA: 1.06 (0.58 to 1.54)<br>ln(PlGF): -1.35 (-1.98 to -0.718)	Max LR+		71%	26.2	0.61	40%	98%
			Max correct		83%	4.3	0.18	86%	80%
			Min LR-		71%	1.9	0.00	100%	46%
UmA category, PlGF & pre-eclampsia[1]	2.66 (0.38 to 4.93)	UmA: 1.11 (0.61 to 1.61)<br>ln(PlGF): -1.18 (-1.81 to -0.55)<br>Pre-eclampsia: 2.38 (0.11 to 4.65)	Max LR+		75%	30.8	0.53	47%	98%
			Max correct		84%	4.5	0.13	89%	80%
			Min LR-		71%	1.9	0.00	100%	46%

[1]n=1 missing from validation set. EDF=end-diastolic flow, PI=pulsatility index, PlGF=placental growth factor, PSG1=pregnancy-specific glycoprotein 1, UmA=umbilical artery. Models generated using the natural log of PlGF in pg/ml but cut points converted back to concentration. Models generated using and centered and scaled values of PSG1. UmA PI centile calculated using Schaffer and Staudach 1997.

Table 10: Details of the validated models to predict development of umbilical artery pulsatility index >95th centile. Constants and coefficients for calculating log(odds) provided, along with variable cut points that give the maximum positive likelihood ratio (LR+), maximum correct classification and minimum negative likelihood ratio (LR-).

Protein(s)	Constant (95% CI)	Coefficient (95% CI)	Cut point	PlGF concentration (pg/ml)	Correctly classified	LR+	LR-	Sensitivity	Specificity
PlGF	4.86 (1.49 to 8.23)	-1.17 (-1.94 to -0.390)	Max LR+	<24	63%	5.9	0.79	24%	96%
			Max correct	<60	74%	3.0	0.38	71%	76%
			Min LR-	<270	59%	1.3	0.00	100%	24%
PlGF & fibronectin	7.09 (1.94 to 12.24)	ln(PlGF): -1.44 (-2.38 to -0.503) Fibronectin: -0.0035 (-0.0091 to 0.0021)	Max LR+		63%	5.6	0.79	24%	96%
			Max correct		76%	3.2	0.31	76%	76%
			Min LR-		61%	1.4	0.00	100%	28%

PlGF=placental growth factor. Models generated using the natural log of PlGF in pg/ml but cut points converted back to concentration.

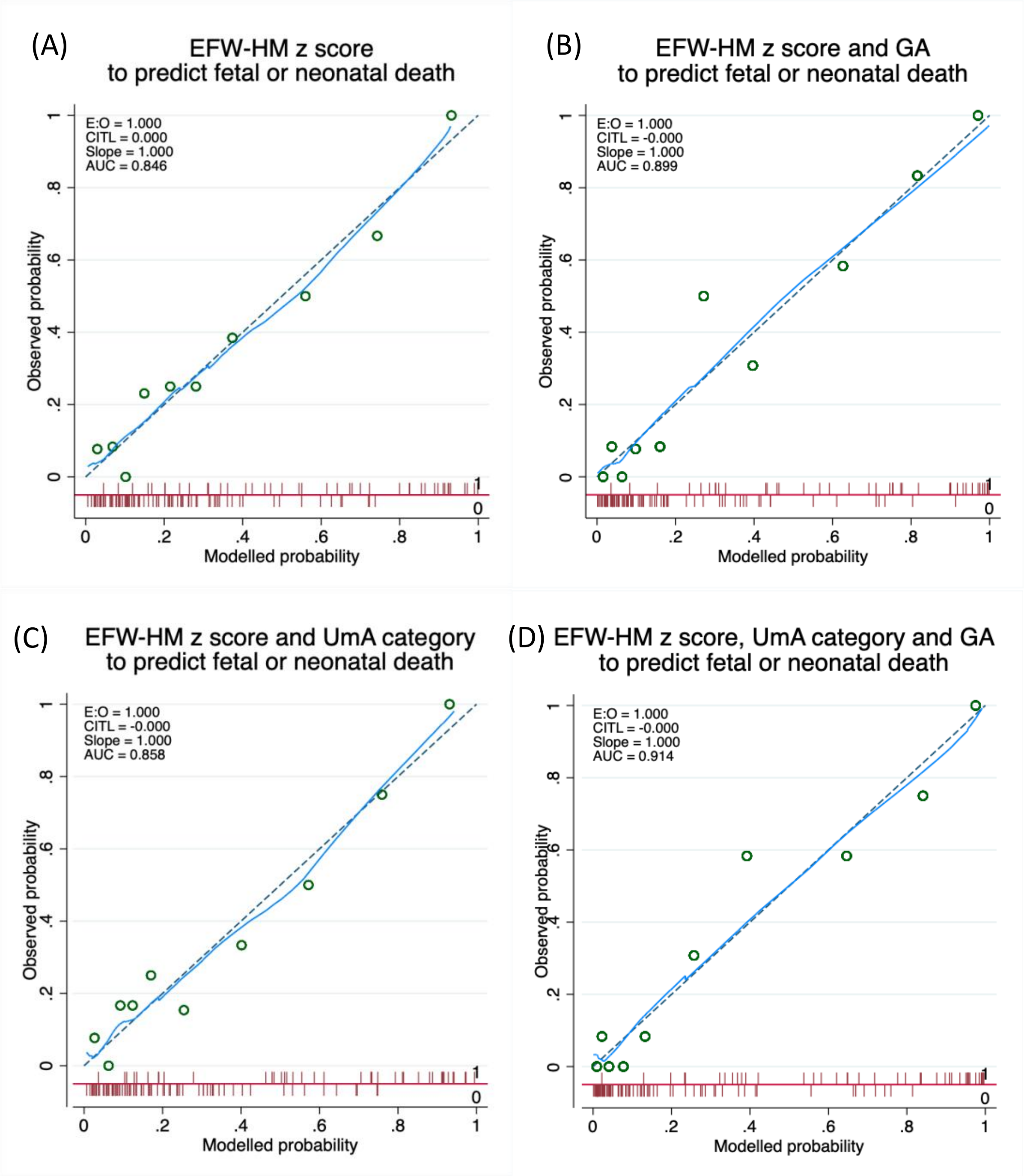

Figure 7: Calibration plots for the models in Tables 8 and 9. Green circles = grouped modelled probability plotted against grouped observed probability, solid blue line = locally weighted scatterplot smoothing (LOWESS), dashed grey line = line of unity. Spike plot (red) at the bottom of the chart area shows distribution of modelled probability by outcome (above line 1=fetal or neonatal death, below line 0=live birth and neonatal survival). CITL=calibration-in-the-large, EFW-HM=estimated fetal weight calculated using Hadlock 3 formula (Hadlock et al., 1985) with z-score calculated using Marsal reference chart (Marsal et al., 1996), E:O=ratio of expected to observed, GA=gestational age at study enrolment, UmA=umbilical artery.

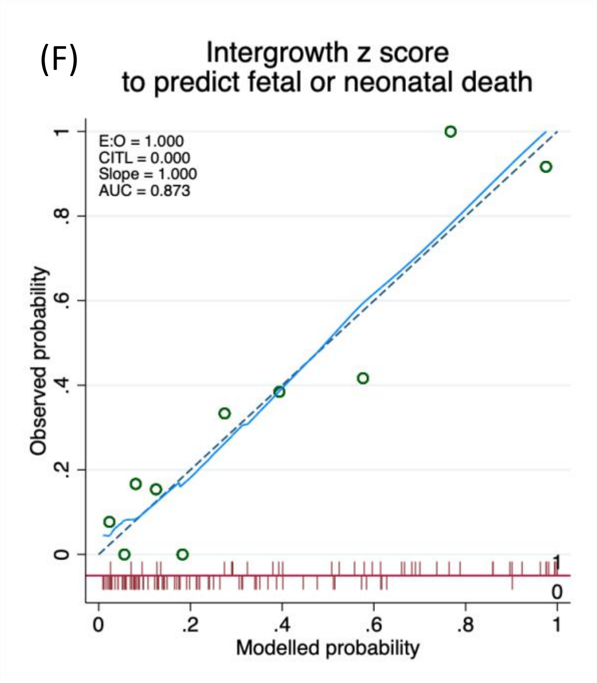

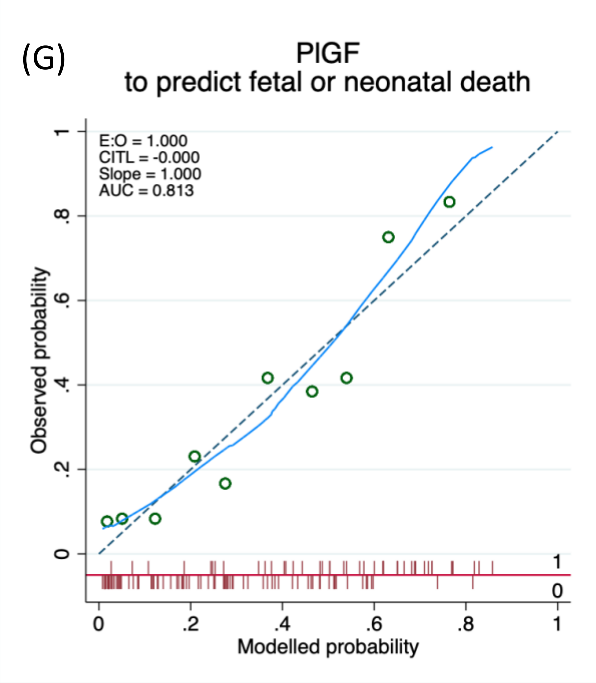

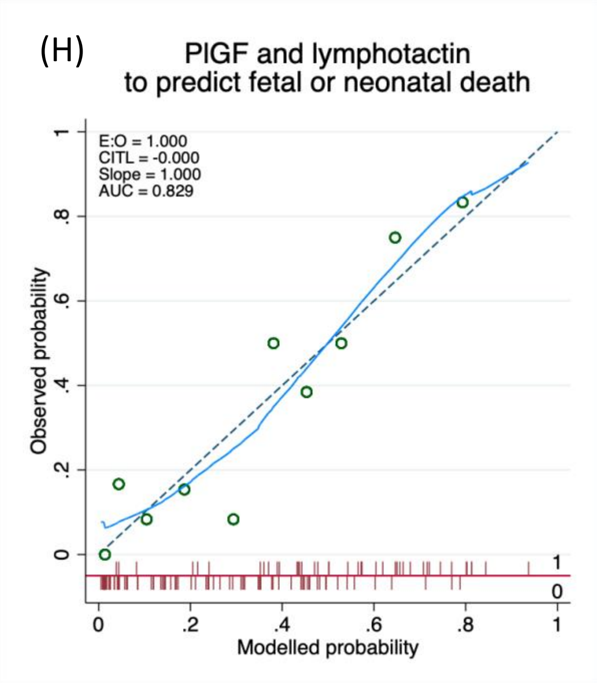

Figure 7 continued

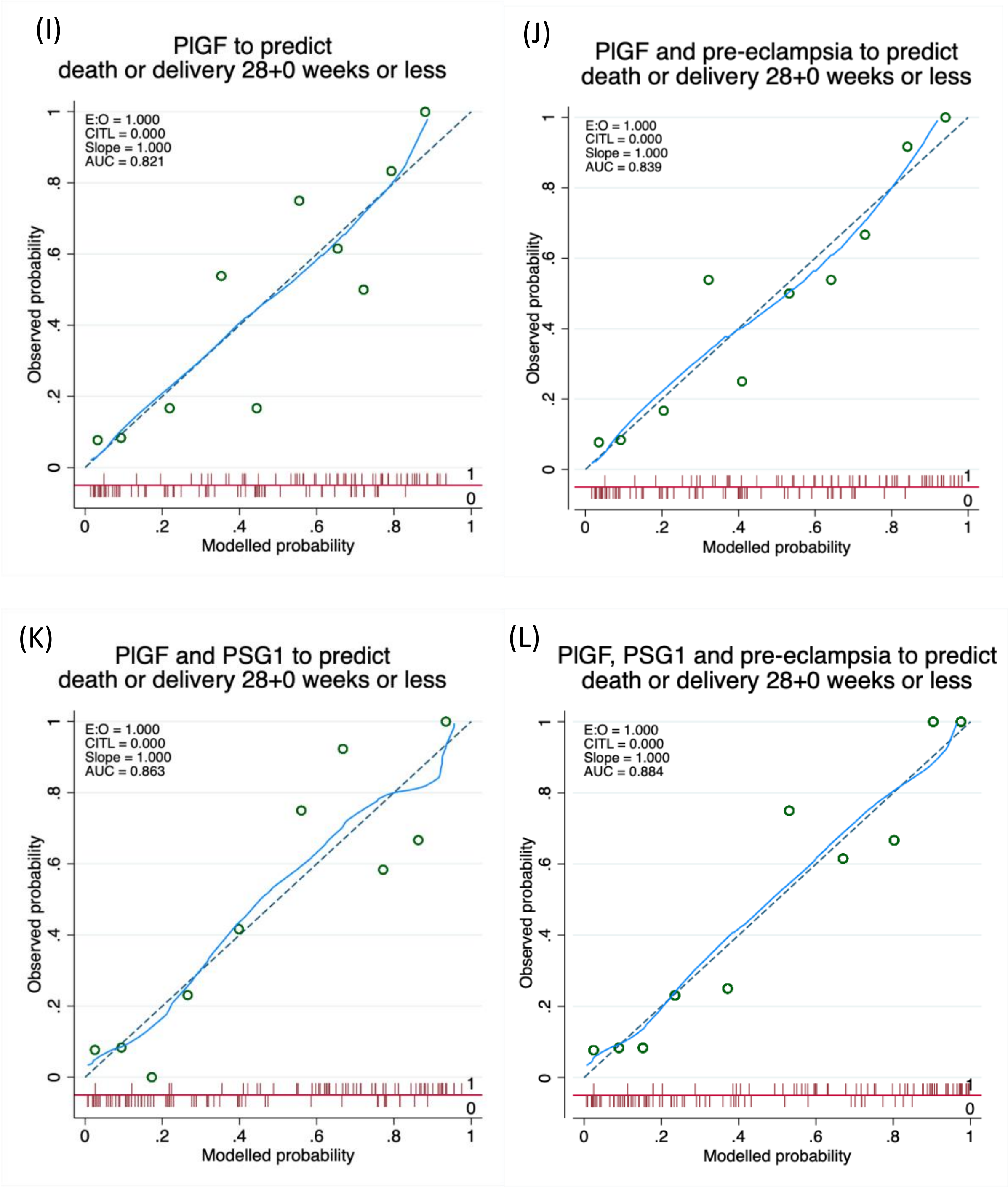

Figure 7 continued

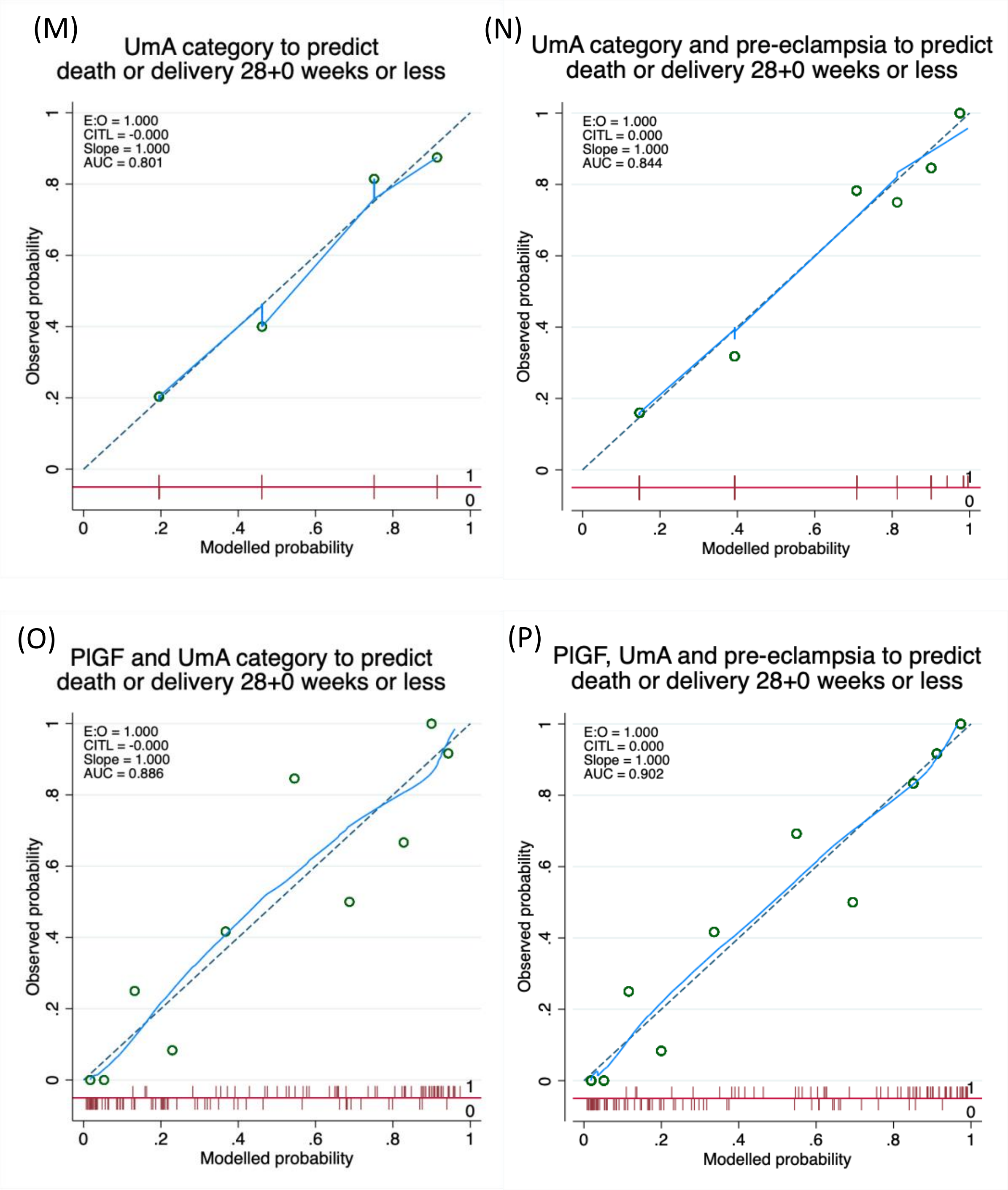

Figure 7 continued

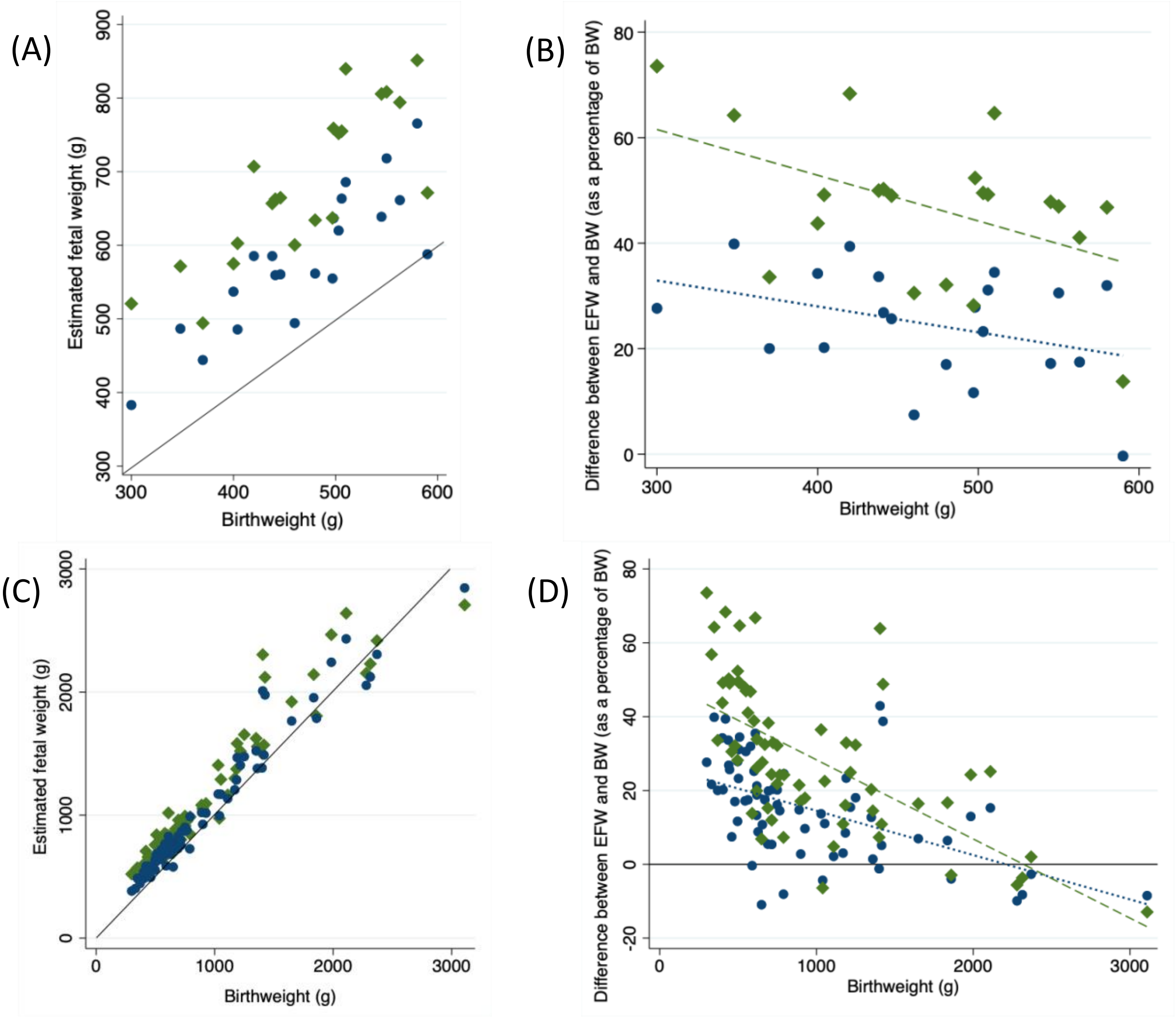

Figure 8: The differences between estimated fetal weights (EFWs) generated using the Intergrowth formula (green diamonds) (Stirnemann et al., 2017) and the Hadlock 3 formula (blue circles) (Hadlock et al., 1985) and birthweight (BW) for livebirths with an EFW performed within seven days of delivery. (A) Absolute differences for n=21 pregnancies with a BW <600g (B) percentage difference, as a percentage of BW, for n=21 pregnancies with a BW <600g (C) absolute differences for all n=67 pregnancies (D) percentage difference, as a percentage of BW, for all n=67 pregnancies. Solid black lines represent EFW=BW, green dashed lines= fitted association between BW and difference between Intergrowth EFW and BW as a percentage of BW, blue dotted line = fitted association between BW and difference between Hadlock 3 EFW and BW as a percentage of BW.

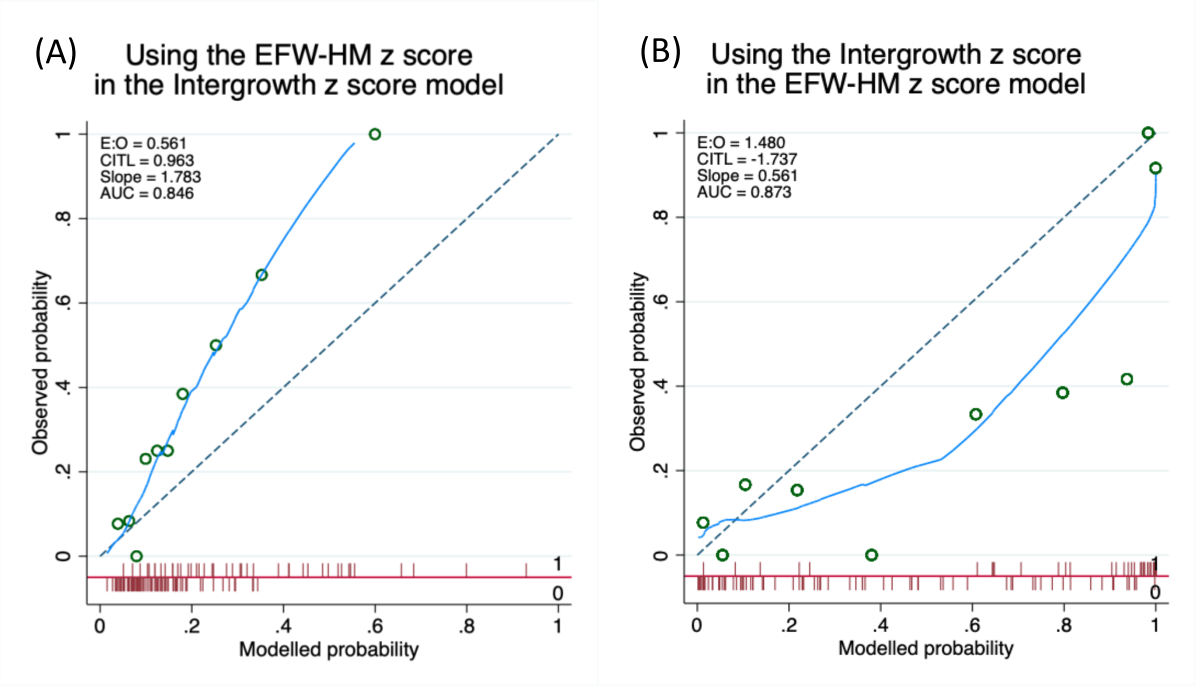

Figure 9: Calibration plots showing (A) the effect of using the estimated fetal weight calculated with the Hadlock 3 formula and z-score from the Marsal reference chart in the model generated from the Intergrowth z-score, and (B) the effect of using the Intergrowth z-score in the model generated from the Hadlock 3 formula estimated fetal weight z-score from the Marsal chart. Green circles = grouped modelled probability plotted against grouped observed probability, solid blue line = locally weighted scatterplot smoothing (LOWESS), dashed grey line = line of unity. Spike plot (red) at the bottom of the chart area shows distribution of modelled probability by outcome (above line 1=fetal or neonatal death, below line 0=live birth and neonatal survival). CITL=calibration-in-the-large, EFW-HM=estimated fetal weight calculated using Hadlock 3 formula (Hadlock et al., 1985) with z-score calculated using Marsal reference chart (Marsal et al., 1996), E:O=ratio of expected to observed. Intergrowth z-score from Stirnemann et al. 2017.

Table 11: Proteins showing an association with pregnancy outcomes at a Benjamini Hochberg 5% false discovery rate and Benjamini Hochberg 1% false discovery rate (over-shaded in blue) in centered and scaled data from the combined discovery and validation sets.

**Protein**	**Abbreviation**	**Fetal or neonatal death -log10(p value)**	**Death or delivery <28+0 weeks of gestation -log10(p value)**
Placental growth factor	PlGF	7.86	10.52
Chorionic somatomammotropin hormone	CSH	6.87	9.84
Pro-adrenomedullin	ADM	4.02	
Pregnancy-specific beta-1 glycoprotein	PSG1	3.42	5.09
Thrombospondin 2	THBS2	3.37	5.04
Hydroxyacid oxidase 1	HAO1	2.94	2.26
Interleukin-17D	IL17D	2.66	2.26
Growth hormone	GH1	2.51	2.38
Macrophage receptor with collagenous structure	MARCO	2.51	
Programmed cell death 1 ligand 2	PDCD1LG2		4.71
Matrix metalloproteinase 12	MMP12		4.31
Soluble FMS-like tyrosine kinase 1	sFLT1		3.31
Galectin-9	LGALS9		3.23
Decorin	DCN		3.20
Interleukin-1 receptor-like 2	IL1RL2		2.99
Carbonic anhydrase 5A	CA5A		2.94
Tumor necrosis factor receptor superfamily member 10B	TNFRSF10B		2.75
CD40 ligand	CD40L		2.59
Fibroblast growth factor 21	FGF21		2.44
Oxidized low density lipoprotein receptor 1	OLR1		2.44
Growth/differentiation factor 2	GDF2		2.30
Tumor necrosis factor receptor superfamily member 10A	TNFRSF10A		2.08
Fibronectin	FN		2.06
Protein AMBP	AMBP		2.05
Follistatin	FST		2.02

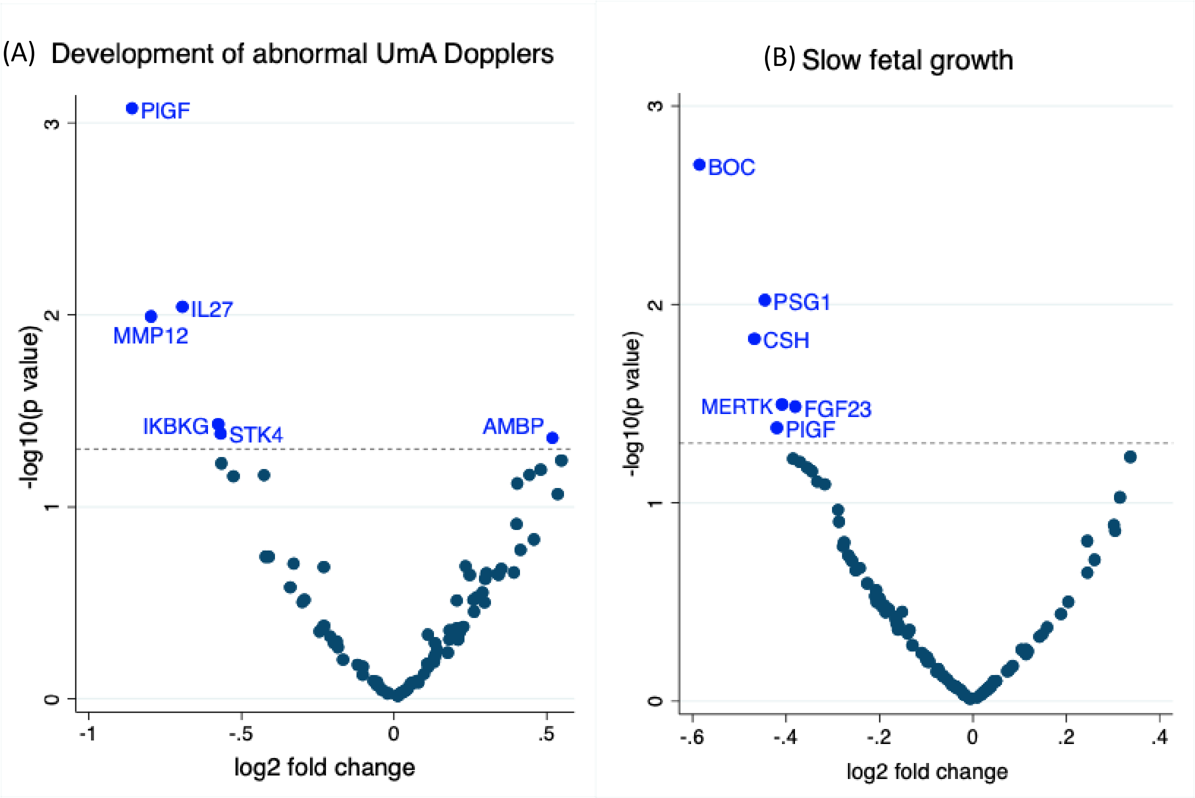

Figure 10: Volcano plots showing the statistical significance and magnitude of associations between the centred and scaled concentrations of the 93 proteins from the discovery and validation sets and (A) the development of abnormal umbilical artery (UmA) Dopplers (pulsatility index >95th centile) (B) slow fetal growth (a worsening of weight deviation of $\geq$10 percentage points over a two-week interval). Associations tested with 2-sided t tests. Dotted line indicates p=0.05. BOC=brother of cysteine dioxygenase, FGF23=fibroblast growth factor 23, CSH=chorionic somatomammotropin hormone, PlGF=placental growth factor, PSG1=pregnancy-specific beta-glycoprotein 1, IL27=interleukin 27, MERTK=tyrosine-protein kinase Mer, MMP12=matrix metalloproteinase-12, IKBKG=inhibitor of nuclear factor kappa B kinase regulatory subunit G, STK4=serine/threonine-protein kinase 4, AMBP=protein AMBP.

Table 12: Top three three-variable and top two-variable models for predicting fetal or neonatal death, death or delivery ≤28+0 weeks of gestation and the development of abnormal umbilical artery (UmA) Dopplers from the combined data set. Leave-one-out cross-validated models generated using centred and scaled values and evaluated on the basis of geometric mean of receiver operating characteristic curve area under the curve (AUC), precision-recall ROC area under the curve (PRROC), Matthews correlation coefficient (MCC) and F_1 rankings, excluding models with variance inflation factors (VIFs) of five or more (see Methods).

Outcome	Variables	Proteins	AUC (95% CI)	PRROC	MCC	F_1
Fetal or neonatal death	3	PlGF, IL16, ADM	0.81 (0.72-0.89)	0.70	0.51	0.65
		ADM, IL17D, PAPPA	0.78 (0.69-0.87)	0.65	0.53	0.63
		ADM, IL17D, PlGF	0.81 (0.73-0.90)	0.68	0.48	0.64
	2	PlGF, sFLT1	0.78 (0.69-0.87)	0.62	0.45	0.59
Death or delivery ≤28+0 weeks of gestation	3	PlGF, CSH, OLR1	0.87 (0.80-0.93)	0.84	0.58	0.78
		PlGF, CSH, MARCO	0.85 (0.79-0.92)	0.83	0.63	0.81
		PlGF, CSH, ADAMTS13	0.86 (0.80-0.93)	0.85	0.50	0.75
	2	PlGF, PSG1	0.84 (0.77-0.92)	0.80	0.57	0.78
Development of abnormal UmA Dopplers	3	PDGFB, PSGL1, IL18	0.79 (0.65-0.94)	0.75	0.60	0.78
		PDGFB, PSGL1, FGF23	0.80 (0.66-0.94)	0.79	0.56	0.75
		PDGFB, CTRC, leptin	0.78 (0.63-0.93)	0.74	0.60	0.78
	2	PDGFB, PSGL1	0.79 (0.64-0.93)	0.73	0.60	0.78

NOTE: the model containing PlGF and sFLT1 is not equivalent to a sFLT1:PlGF ratio. ADAMTS13=A disintegrin and metalloproteinase with thrombospondin motifs 13, ADM=pro-adrenomedullin, CTRC=chymotrypsin C, FGF23=fibroblast growth factor 23, CSH= chorionic somatomammotropin hormone, OLR1=oxidised low density lipoprotein receptor 1, MARCO=macrophage receptor with collagenous structure, PAPPA=pregnancy-associated plasma protein A, PDGFB=platelet-derived growth factor subunit B, PlGF=placental growth factor, PSG1=pregnancy-specific beta-1 glycoprotein, PSGL1=p-selectin glycoprotein ligand 1, sFLT1=soluble fms-like tyrosine kinase 1.

Table 13: Protein and ultrasound measurements at enrolment showing a significant association with gestational age at livebirth or diagnosis of fetal death and/or interval between enrolment and livebirth or diagnosis of fetal death at a Benjamini-Hochberg 1% false discovery rate.

	Strength of association with gestational age at livebirth or diagnosis of fetal death -log10(p value)	Strength of association with interval between enrolment and livebirth or diagnosis of fetal death or livebirth -log10(p value)
PlGF	22.0	17.2
CSH	11.1	8.0
UmA category	9.9	11.0
Mean UtA PI	7.2	6.5
Thrombospondin 2	7.1	5.5
sFLT1	6.9	8.0
MMP12	6.1	6.8
Decorin	5.7	4.7
IL1RL2	5.1	
PSG1	5.1	
Growth hormone	4.2	
Protein AMBP	4.2	

CSH= chorionic somatomammotropin hormone, IL1RL2=interleukin-1 receptor-like 2, MMP12=matrix metalloproteinase 12, PlGF=placental growth factor, PSG1=pregnancy specific glycoprotein 1, sFLT1=soluble fms-like tyrosine kinase 1, UmA=umbilical artery, UtA PI=uterine artery pulsatility index.

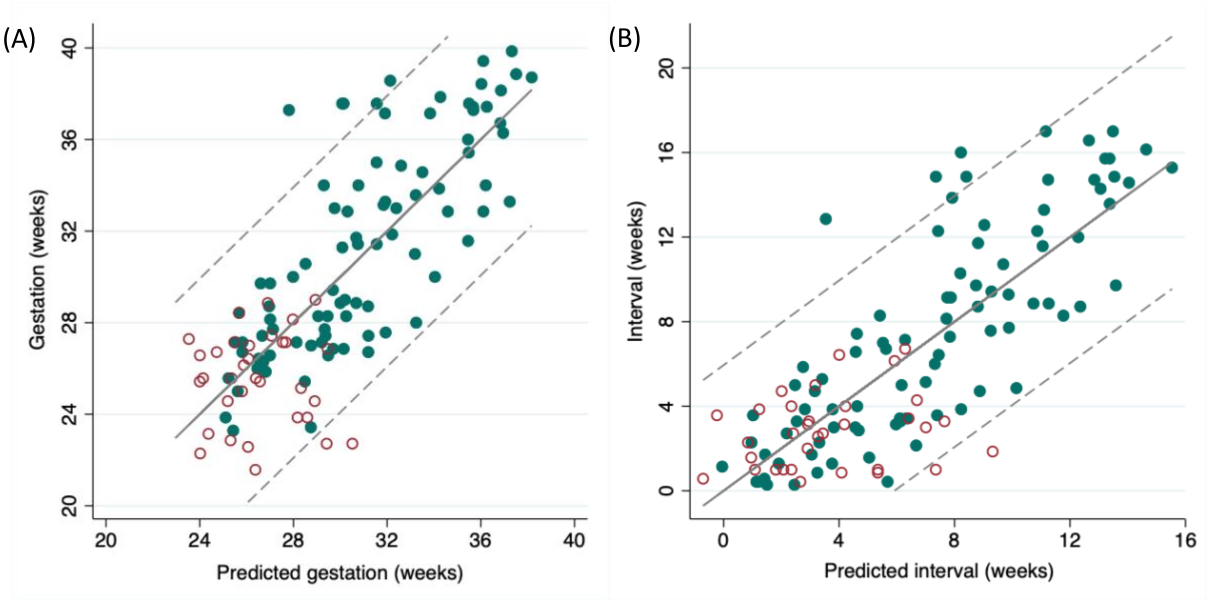

Figure 11: (A) Predicted versus actual gestational age of either livebirth or diagnosis of fetal death, based on the sparser model containing PlGF and sFLT1 concentration and umbilical artery Doppler category (B) Predicted versus actual interval from enrolment to either livebirth or diagnosis of fetal death, based on the sparser model containing PlGF and sFLT1 concentrations, umbilical artery Doppler category and gestational age at enrolment. Green filled circles=pregnancies ending in livebirth, red hollow circles=pregnancies ending in fetal death, dotted lines=95% prediction intervals.

Table 14: Comparison of the maternal and pregnancy characteristics of participants with and without samples available for placental histological classification.

Characteristics and outcomes	Sample available (n=55)	No sample available (n=68)	p value
Maternal age (years, mean SD)	33 (6.7)	34 (5.9)	0.18[1]
BMI (median IQR)	24.9 (22.8-29.0)	26.2 (22.8-30.0)	0.87[2]
Ethnicity (n %)			
White	33 (61)	37 (56)	0.82[3]
Black	11 (20)	14 (21)	
Asian	10 (18)	15 (23)	
Mean UtA PI >95th centile at enrolment (Schaffer and Staudach, 1997) (n %)	43 (80)	55 (81)	0.86[3]
Female fetus (n %)	34 (63)	26 (41)	0.016[3]
Stillbirth (n %)	14 (25)	19 (28)	0.76[3]
Gestational age at delivery (median IQR)	28+3 (26+5 to 33+0)	27+6 (26+2 to 34+1)	0.81[2]
Caesarean delivery (n %)	40 (73)	39 (58)	0.10[3]

PI=pulsatility index, UmA=umbilical artery, UtA=uterine artery. [1]two-sided t test, [2]Wilcoxon rank sum, [3]chi square test. Missing: maternal ethnicity for 1 pregnancy with a placental sample and two pregnancies without placental samples; mean UtA PI for one pregnancy with a placental sample; fetal sex for one stillborn pregnancy with a placental sample; fetal sex for four stillborn pregnancies without placental samples.

Table 15: Pregnancy outcome by Amsterdam consensus placental classification (Khong et al., 2016) for the 55 pregnancies with available placental histology samples.

Classification		Stillbirth	Neonatal death	Neonatal survival
Normal	n=10	1	0	9
MVM	n=39	12	0	27
VUE	n=3	1	1	1
Dysmorphic villi	n=2	0	1	1
FVM	n-1	0	1	0

FVM=fetal vascular malperfusion, MVM=maternal vascular malperfusion, VUE=villitis of unknown aetiology.

Table 16: Associations between ultrasound measurements and maternal serum protein concentrations at enrolment and subsequent placental classification of (1) any placental pathology (2) maternal vascular malperfusion (MVM) according to the Amsterdam consensus criteria (Khong et al., 2016).

		Any placental pathology (n=45)	No placental pathology identified (n=10)	p value	MVM (n=39)	non-MVM (n=16)	p value	AUC (95% CI)
EFW_{HM} z-score (median IQR) (Hadlock et al., 1985, Marsal et al., 1996)		-3.0 (-3.4 to -2.6)	-2.8 (-3.4 to -2.0)	0.44	-2.9 (-3.3 to -2.6)	-3.2 (-3.4 to -2.6)	0.63	
EFW_{Int} z-score (median IQR) (Stirnemann et al., 2017)		-3.4 (-4.3 to -2.6)	-3.0 (-4.9 to -2.6)	0.72	-3.4 (-4.2 to -2.6)	-3.4 (-4.9 to -2.6)	0.78	
UmA category at enrolment (n %)	PI $\leq 95^{th}$ centile	19 (42)	6 (60)	0.57[#]	18 (46)	7 (44)	0.54[#]	
	PI $>95^{th}$ centile with positive EDF	14 (31)	1 (10)		12 (31)	3 (19)		
	Absent EDF	6 (13)	2 (20)		4 (10)	4 (25)		
	Reversed EDF	6 (13)	1 (10)		5 (13)	2 (13)		
Abnormal UmA PI ($>95^{th}$ centile) at any point before delivery (n %) (Schaffer and Staudach, 1997)		33 (73)	5 (50)	0.15	28 (72)	10 (63)	0.50	
Absent or reversed UmA end-diastolic flow at any point before delivery (n %)		27 (60)	4 (40)	0.30[#]	22 (56)	9 (56)	0.99	
Mean UtA PI at enrolment (mean SD)[1]		1.70 (0.67)	1.59 (0.69)	0.63	1.80 (0.63)	1.40 (0.68)	0.044	0.68 (0.51-0.84)
PlGF (pg/ml; median IQR)		27.8 (18.0-56.6)	73.3 (22.6-279.6)	0.06	26.7 (17.9-56.6)	61.7 (29.8-195.8)	0.043	0.68 (0.52-0.84)
CSH (mcg/ml; median IQR)		110 (77-141)	134 (85-186)	0.49	101 (74-140)	135 (93-182)	0.24	
PAPPA (NPX; mean SD)		0.15 (0.88)	-0.28 (0.71)	0.16	0.23 (0.76)	-0.31 (1.01)	0.036	0.67 (0.51-0.84)

EFW_{HM}=estimated fetal weight calculated using Hadlock 3 formula (Hadlock et al., 1985) with z-score calculated using Marsal reference chart (Marsal et al., 1996), EFW_{Int}=estimated fetal weight and z-score calculated using Intergrowth formula and chart (Stirnemann et al., 2017), CSH= chorionic somatomammotropin hormone, NPX=normalised protein expression, PAPPA=pregnancy-associated plasma protein A, PI=pulsatility index, PlGF=placental growth factor, UmA=umbilical artery, UtA=uterine artery. PLGF analysed as a natural log for AUC, NPX centred and scaled. n=1 missing from MVM, [#]Fisher's exact test

Methods

Equation 1: Hadlock 3 EFW formula (Hadlock et al., 1985)

$$log_{10}EFW = 1.326 - 0.00326 \times AC \times FL + 0.0107 \times HC + 0.0438 \times AC + 0.158 \times FL$$

Where AC = abdominal circumference, FL = femur length and HC = head circumference. EFW given in grams with measurements in centimetres.

Equation 2: Marsal EFW z-score (Marsal et al., 1996)

$$z\ score = \frac{EFW - \left(-2.278843 \times 10^{-6} {GA_d}^4 + 1.402168 \times 10^{-3} {GA_d}^3 - 2.008726 \times 10^{-1} {GA_d}^2 + 9.284121 GA_d - 41.25956\right)}{0.12\left(-2.278843 \times 10^{-6} {GA_d}^4 + 1.402168 \times 10^{-3} {GA_d}^3 - 2.008726 \times 10^{-1} {GA_d}^2 + 9.284121 GA_d - 41.25956\right)}$$

Where GA_d = gestational age in days

Equation 3: Percentage weight deviation (Marsal, 2009)

$$weight\ deviation = \frac{(EFW - 50th\ centile\ fetal\ weight\ for\ gestation)}{50th\ centile\ fetal\ weight\ for\ gestation} \times 100$$

Where 50th centile fetal weights for gestation are calculated using the formula for Marsal z-score with a z-score of 0.

Equation 4: Intergrowth EFW formula (Stirnemann et al., 2017)

$$\log(EFW) = 5.08482 - 54.06633 \times \left(\frac{AC}{100}\right)^3 - 95.80076 \times \left(\frac{AC}{100}\right)^3 \times \log\left(\frac{AC}{100}\right) + 3.136370 \times (\frac{HC}{100})$$

Where AC = abdominal circumference and HC = head circumference. EFW given in grams with measurements in centimetres.

Equation 5: Intergrowth z-score formula (Stirnemann et al., 2017)

$$\lambda(GA) = -4.257629 - 2162.234 \times GA^{-2} + 0.0002301829 \times GA^3$$
$$\mu(GA) = 4.956737 + 0.0005019687 \times GA^3 - 0.0001227065 \times GA^3 \times \log(GA)$$
$$\sigma(GA) = 10^{-4} \times (-6.997171 + 0.057559 \times GA^3 - 0.01493946 \times GA^3 \times \log(GA))$$

If λ(GA) = 0

$$z\ score = \sigma(GA)^{-1} \times \log\left(\frac{Y}{\mu(GA)}\right)$$

If λ(GA) ≠ 0

$$z\ score = (\sigma(GA) \times \lambda(GA))^{-1} \times \left(\left(\frac{Y}{\mu(GA)}\right)^{\lambda(GA)} - 1\right)$$

Where λ(GA) = skewness for given gestational age, GA = exact gestational age in weeks, μ(GA) = mean EFW for given gestational age, σ(GA) = coefficient of variation for given gestational age, Y = log(EFW), all logs are natural logs.

Table 17: Full list of proteins measured by the Olink Proseek® Multiplex cardiovascular disease (CVD) II proximity extension assay with reported intra-assay variation. Note PlGF is included in the panel, but the PlGF normalised protein expression (NPX) values were not used for our analysis. Instead, we used PlGF concentration, as measured by Elecsys® electrochemiluminescence immunoassays (Roche Diagnostics).

**Protein name (abbreviation)**	**UniProt No**	**Intra-assay variation**
Pro-adrenomedullin (ADM)	P35318	13%
2,4-dienoyl-CoA reductase, mitochondrial (DECR1)	Q16698	15%
A disintegrin and metalloproteinase with thrombospondin motifs 13 (ADAMTS13)	Q76LX8	4.0%
Agouti-related protein (AGRP)	O00253	8.6%
Alpha-L-iduronidase (IDUA)	P35475	6.5%
Angiopoietin 1 (ANGPT1)	Q15389	8.8%
Angiopoietin 1 receptor (TIE2)	Q02763	7.7%
Angiotensin-converting enzyme 2 (ACE2)	Q9BYF1	7.9%
Bone morphogenetic protein 6 (BMP6)	P22004	21%
Brother of cysteine dioxygenase (BOC)	Q9BWV1	9.6%
Carbonic anhydrase 5A, mitochondrial (CA5A)	P35218	8.8%
Carcinoembryonic antigen related cell adhesion molecule 8 (CEACAM8)	P31997	11%
Cathepsin L1 (CTSL1)	P07711	10%
C-C motif chemokine 17 (CCL17)	Q92583	12%
C-C motif chemokine 3 (CCL3)	P10147	8.6%
CD40 ligand (CD40L)	P29965	9.1%
Chymotrypsin C (CTRC)	Q99895	9.8%
C-X-C motif chemokine 1 (CXCL1)	P09341	9.6%
Decorin (DCN)	P07585	7.5%
Dickkopf-related protein 1 (DKK1)	O94907	11%

Fatty acid-binding protein, intestinal (FABP2)	P12104	8.5%
Fibroblast growth factor 21 (FGF21)	Q9NSA1	12%
Fibroblast growth factor 23 (FGF23)	Q9GZV9	14%
Follistatin (FST)	P19883	9.3%
Galectin 9 (LGALS9)	O00182	5.2%
Gastric intrinsic factor (GIF)	P27352	11%
Gastrotropin (GT)	P51161	16%
Growth hormone (GH1)	P01241	7.4%
Growth/differentiation factor 2 (GDF2)	Q9UK05	8.8%
Heat shock 27 kDa protein (HSP27)	P04792	11%
Heme oxygenase 1 (HO1)	P09601	8.1%
Hydroxyacid oxidase 1 (HAO1)	Q9UJM8	8.7%
Interleukin 1 receptor antagonist protein (IL1RA)	P18510	12%
Interleukin 1 receptor-like 2 (IL1RL2)	Q9HB29	10%
Interleukin 17D (IL17D)	Q8TAD2	13%
Interleukin 18 (IL18)	Q14116	11%
Interleukin 27 (IL27)	Q8NEV9<br>Q14213	7.2%
Interleukin 4 receptor subunit alpha (IL4RA)	P24394	9.2%
Interleukin 6 (IL6)	P05231	8.8%
Hepatitis A virus cellular receptor 1 (HAVCR1)	Q96D42	11%
Lactoylglutathione lyase (GLO1)	Q04760	8.3%
Oxidised low density lipoprotein receptor 1 (OLR1)	P78380	9.0%
Leptin (LEP)	P41159	6.2%
Lipoprotein lipase (LPL)	P06858	7.2%
Low affinity immunoglobulin gamma Fc region receptor II-b (FCGR2B)	P31994	9.1%
Lymphotactin (XCL1)	P47992	10%
Macrophage receptor with collagenous structure (MARCO)	Q9UEW3	6.1%
Matrix metalloproteinase 12 (MMP12)	P39900	11%
Matrix metalloproteinase 7 (MMP7)	P09237	8.5%
Melusin (ITGB1BP2)	Q9UKP3	11%
Natriuretic peptides B (BNP)	P16860	-
Inhibitor of nuclear factor kappa B kinase regulatory subunit G (IKBKG)	Q9Y6K9	9.1%
Osteoclast-associated immunoglobulin-like receptor (OSCAR)	Q8IYS5	4.9%
Pappalysin 1 (PAPPA)	Q13219	13%
Pentraxin-related protein PTX3 (PTX3)	P26022	8.1%
Placenta growth factor (PlGF)	P49763	12%
Platelet-derived growth factor subunit B (PDGFB)	P01127	11%
Poly [ADP-ribose] polymerase 1 (PARP1)	P09874	9.4%
Polymeric immunoglobulin receptor (PIGR)	P01833	3.4%
Programmed cell death 1 ligand 2 (PDCD1LG2)	Q9BQ51	8.7%
Heparin binding EGF-like growth factor (HBEGF)	Q99075	8.4%
Pro-interleukin 16 (IL16)	Q14005	11%
Prolargin (PRELP)	P51888	6.9%
Prostasin (PRSS8)	Q16651	7.6%
Protein AMBP (AMBP)	P02760	5.6%
Proteinase-activated receptor 1 (PAR1)	P25116	9.4%
Transglutaminase 2 (TGM2)	P21980	8.0%
Proto-oncogene tyrosine-protein kinase SRC (SRC)	P12931	9.7%
P-selectin glycoprotein ligand 1 (PSGL1)	Q14242	5.9%

Receptor for advanced glycosylation end products (RAGE)	Q15109	8.8&
Renin (REN)	P00797	7.8%
Serine protease 27 (PRSS27)	Q9BQR3	9.3%
Serine/threonine-protein kinase 4 (STK4)	Q13043	7.2%
Serpin A12 (SERPINA12)	Q8IW75	10%
SLAM family member 5 (CD84)	Q9UIB8	9.0%
SLAM family member 7 (SLAMF7)	Q9NQ25	11%
Sortilin 1 (SORT1)	Q99523	7.8%
Spondin 2 (SPON2)	Q9BUD6	5.1%
Stem cell factor (SCF)	P21583	7.3%
Superoxide dismutase [Mn], mitochondrial (SOD2)	P04179	5.9%
T-cell surface glycoprotein CD4 (CD4)	P01730	9.6%
Thrombomodulin (TM)	P07204	11%
Thrombopoietin (THPO)	P40225	9.3%
Thrombospondin 2 (THBS2)	P35442	5.4%
Tissue factor (TF)	P13726	8.2%
Tumor necrosis factor receptor superfamily member 10B (TNFRSF10B)	O14763	10%
Tumor necrosis factor receptor superfamily member 10A (TNFRSF10A)	O00220	11%
Tumor necrosis factor receptor superfamily member 11A (TNFRSF11A)	Q9Y6Q6	10%
Tumor necrosis factor receptor superfamily member 13B (TNFRSF13B)	O14836	9.5%
Tyrosine-protein kinase Mer (MERTK)	Q12866	10%
Vascular endothelial growth factor D (VEGFD)	O43915	7.2%
V-set and immunoglobulin domain-containing protein 2 (VSIG2)	Q96IQ7	8.2%

Table 18: The scoring system for ranking proteins identified through pooled mass spectrometry, using the following formula:

$$Score = \sum_{i=1}^{4} r_i v_i + t + p + u$$

Category	Criteria	Score
Ratio score (r)	>1.33 or <0.75	5
	>1.5 or <0.67	10
	>2 or <0.5	15
	>4 or <0.25	20
Variability score (v)	>50%	0
	31 to 50%	1
	16 to 30%	1.34
	≤15%	1.50
Trend score (t)	2 ratios both >1.2 or both <0.83	5
	3 ratios all >1.2 or all <0.83	10
	4 ratios all >1.2 or all <0.83	20
Peptide score (p)	1	-50
	2-4	0
	≥5	5
Ubiquity score (u)	One of the 12 depleted proteins	-50
	Haemoglobin protein	-20

Table 19: Top 50 highest scoring proteins identified from pooled serum liquid chromatography and tandem mass spectrometry, based on the scoring system above.

UniProt code	Description
P02751	Fibronectin OS=Homo sapiens GN=FN1 PE=1 SV=4 - [FINC_HUMAN]
P02741	C-reactive protein OS=Homo sapiens GN=CRP PE=1 SV=1 - [CRP_HUMAN]
P0DJI9	Serum amyloid A-2 protein OS=Homo sapiens GN=SAA2 PE=1 SV=1 - [SAA2_HUMAN]
P20848	Putative alpha-1-antitrypsin-related protein OS=Homo sapiens GN=SERPINA2P PE=5 SV=1 - [A1ATR_HUMAN]
P0DML2	Chorionic somatomammotropin hormone 1 OS=Homo sapiens GN=CSH1 PE=1 SV=1 - [CSH1_HUMAN]
P11464	Pregnancy-specific beta-1-glycoprotein 1 OS=Homo sapiens GN=PSG1 PE=2 SV=1 - [PSG1_HUMAN]
Q13046	Putative pregnancy-specific beta-1-glycoprotein 7 OS=Homo sapiens GN=PSG7 PE=5 SV=2 - [PSG7_HUMAN]
Q00887	Pregnancy-specific beta-1-glycoprotein 9 OS=Homo sapiens GN=PSG9 PE=2 SV=2 - [PSG9_HUMAN]
P01591	Immunoglobulin J chain OS=Homo sapiens GN=IGJ PE=1 SV=4 - [IGJ_HUMAN]
P0DJI8	Serum amyloid A-1 protein OS=Homo sapiens GN=SAA1 PE=1 SV=1 - [SAA1_HUMAN]
P01834	Ig kappa chain C region OS=Homo sapiens GN=IGKC PE=1 SV=1 - [IGKC_HUMAN]
Q6N069	N-alpha-acetyltransferase 16, NatA auxiliary subunit OS=Homo sapiens GN=NAA16 PE=1 SV=2 - [NAA16_HUMAN]
Q8TDD5	Mucolipin-3 OS=Homo sapiens GN=MCOLN3 PE=1 SV=1 - [MCLN3_HUMAN]
Q9UQ72	Pregnancy-specific beta-1-glycoprotein 11 OS=Homo sapiens GN=PSG11 PE=2 SV=3 - [PSG11_HUMAN]
Q16557	Pregnancy-specific beta-1-glycoprotein 3 OS=Homo sapiens GN=PSG3 PE=2 SV=2 - [PSG3_HUMAN]
Q00888	Pregnancy-specific beta-1-glycoprotein 4 OS=Homo sapiens GN=PSG4 PE=2 SV=3 - [PSG4_HUMAN]
P02760	Protein AMBP OS=Homo sapiens GN=AMBP PE=1 SV=1 - [AMBP_HUMAN]
Q9UIQ6	Leucyl-cystinyl aminopeptidase OS=Homo sapiens GN=LNPEP PE=1 SV=3 - [LCAP_HUMAN]
P0CG05	Ig lambda-2 chain C regions OS=Homo sapiens GN=IGLC2 PE=1 SV=1 - [LAC2_HUMAN]
P55196	Afadin OS=Homo sapiens GN=MLLT4 PE=1 SV=3 - [AFAD_HUMAN]
P04196	Histidine-rich glycoprotein OS=Homo sapiens GN=HRG PE=1 SV=1 - [HRG_HUMAN]
P04275	von Willebrand factor OS=Homo sapiens GN=VWF PE=1 SV=4 - [VWF_HUMAN]
Q06033	Inter-alpha-trypsin inhibitor heavy chain H3 OS=Homo sapiens GN=ITIH3 PE=1 SV=2 - [ITIH3_HUMAN]
Q13219	Pappalysin-1 OS=Homo sapiens GN=PAPPA PE=1 SV=3 - [PAPP1_HUMAN]
O95445	Apolipoprotein M OS=Homo sapiens GN=APOM PE=1 SV=2 - [APOM_HUMAN]
Q9UN37	Vacuolar protein sorting-associated protein 4A OS=Homo sapiens GN=VPS4A PE=1 SV=1 - [VPS4A_HUMAN]
P11226	Mannose-binding protein C OS=Homo sapiens GN=MBL2 PE=1 SV=2 - [MBL2_HUMAN]
O75636	Ficolin-3 OS=Homo sapiens GN=FCN3 PE=1 SV=2 - [FCN3_HUMAN]
P48740	Mannan-binding lectin serine protease 1 OS=Homo sapiens GN=MASP1 PE=1 SV=3 - [MASP1_HUMAN]
O14791	Apolipoprotein L1 OS=Homo sapiens GN=APOL1 PE=1 SV=5 - [APOL1_HUMAN]
O75882	Attractin OS=Homo sapiens GN=ATRN PE=1 SV=2 - [ATRN_HUMAN]

P00450	Ceruloplasmin OS=Homo sapiens GN=CP PE=1 SV=1 - [CERU_HUMAN]
P00734	Prothrombin OS=Homo sapiens GN=F2 PE=1 SV=2 - [THRB_HUMAN]
P00736	Complement C1r subcomponent OS=Homo sapiens GN=C1R PE=1 SV=2 - [C1R_HUMAN]
P00740	Coagulation factor IX OS=Homo sapiens GN=F9 PE=1 SV=2 - [FA9_HUMAN]
P00742	Coagulation factor X OS=Homo sapiens GN=F10 PE=1 SV=2 - [FA10_HUMAN]
P00747	Plasminogen OS=Homo sapiens GN=PLG PE=1 SV=2 - [PLMN_HUMAN]
P00748	Coagulation factor XII OS=Homo sapiens GN=F12 PE=1 SV=3 - [FA12_HUMAN]
P00751	Complement factor B OS=Homo sapiens GN=CFB PE=1 SV=2 - [CFAB_HUMAN]
P01008	Antithrombin-III OS=Homo sapiens GN=SERPINC1 PE=1 SV=1 - [ANT3_HUMAN]
P01011	Alpha-1-antichymotrypsin OS=Homo sapiens GN=SERPINA3 PE=1 SV=2 - [AACT_HUMAN]
P01019	Angiotensinogen OS=Homo sapiens GN=AGT PE=1 SV=1 - [ANGT_HUMAN]
P01024	Complement C3 OS=Homo sapiens GN=C3 PE=1 SV=2 - [CO3_HUMAN]
P01031	Complement C5 OS=Homo sapiens GN=C5 PE=1 SV=4 - [CO5_HUMAN]
P01042	Kininogen-1 OS=Homo sapiens GN=KNG1 PE=1 SV=2 - [KNG1_HUMAN]
P02649	Apolipoprotein E OS=Homo sapiens GN=APOE PE=1 SV=1 - [APOE_HUMAN]
P02654	Apolipoprotein C-I OS=Homo sapiens GN=APOC1 PE=1 SV=1 - [APOC1_HUMAN]
P02655	Apolipoprotein C-II OS=Homo sapiens GN=APOC2 PE=1 SV=1 - [APOC2_HUMAN]
P02656	Apolipoprotein C-III OS=Homo sapiens GN=APOC3 PE=1 SV=1 - [APOC3_HUMAN]
P02743	Serum amyloid P-component OS=Homo sapiens GN=APCS PE=1 SV=2 - [SAMP_HUMAN]

Table 20: Combinations of proteins included in different sections of the study

Platform	Protein(s)	Tested in discovery set	Univariate association with outcomes in discovery set	Model generation from discovery set	Parenclitic network analysis	Tested in validation set	Univariate association with outcomes in combined set
Roche Elecsys	PlGF & sFLT1	✓	✓	✓	✓	✓	✓
ELISA	VEGFA	✓			✓		
	VEGFD, endoglin, NRP1, VEGFR2	✓	✓	✓	✓		
	FN, CSH, PSG1	✓	✓	✓	✓	✓	✓
	LNPEP & SAA	✓	✓	✓	✓		
Olink multiplex	PlGF	✓				✓	
	VEGFD	✓				✓	✓
	BNP, melusin, PARP1	✓			✓	✓	
	Remaining 87 proteins	✓	✓	✓	✓	✓	✓
Totals			98	98	102		93

Table 21: Marker priority survey questions for clinicians and patients. Importance was ranked on the following 5-point Likert scale: very important; important; moderately important; slightly important; not important.

Stakeholder	Question area	Full question wording
Clinicians	Importance of outcomes for providing clinical care	How important is predicting which pregnancies will end in fetal or neonatal death for providing clinical care?
		When providing clinical care, which is more important: correctly identifying women who WILL have this outcome (true positives, sensitivity) or correctly identifying women who will NOT have this outcome (true negatives, specificity)?
		How important is predicting which pregnancies will end in fetal death or the need for delivery before 28 weeks of gestation for providing clinical care?
		When providing clinical care, which is more important: correctly identifying women who WILL have this outcome (true positives, sensitivity) or correctly identifying women who will NOT have this outcome (true negatives, specificity)?
		How important is predicting that umbilical artery Dopplers would become abnormal (pulsatility index >95th centile) if they were normal at the time of diagnosis for providing clinical care?
		When providing clinical care, which is more important: correctly identifying women who WILL have this outcome (true positives, sensitivity) or correctly identifying women who will NOT have this outcome (true negatives, specificity)?
		How important is predicting which pregnancies will end in a live birth at 37+0 or later for providing clinical care?
		When providing clinical care, which is more important: correctly identifying women who WILL have this outcome (true positives, sensitivity) or correctly identifying women who will NOT have this outcome (true negatives, specificity)?
	Importance of outcomes for patient counselling	How important is predicting which pregnancies will end in fetal or neonatal death for patient counselling?
		When counselling patient, which is more important: correctly identifying women who WILL have this outcome (true positives, sensitivity) or correctly identifying women who will NOT have this outcome (true negatives, specificity)?
		How important is predicting which pregnancies will end in fetal death or the need for delivery before 28 weeks of gestation for patient counselling?
		When counselling patient, which is more important: correctly identifying women who WILL have this outcome (true positives, sensitivity) or correctly identifying women who will NOT have this outcome (true negatives, specificity)?
		How important is predicting that umbilical artery Dopplers would become abnormal (pulsatility index >95th centile) if they were normal at the time of diagnosis for patient counselling?
		When counselling patient, which is more important: correctly identifying women who WILL have this outcome (true positives, sensitivity) or correctly identifying women who will NOT have this outcome (true negatives, specificity)?
		How important is predicting which pregnancies will end in a live birth at 37+0 or later for patient counselling?
		When counselling patient, which is more important: correctly identifying women who WILL have this outcome (true positives, sensitivity) or correctly identifying women who will NOT have this outcome (true negatives, specificity)?

Stakeholder	Full question wording
Patients	How important is it to be able to predict whether the pregnancy will end in the death of the baby, either inside the womb or after birth (fetal or neonatal death)?
	Which is more important: correctly identifying women who WILL have this outcome (true positives, sensitivity) or correctly identifying women who will NOT have this outcome (true negatives, specificity)?
	How important is it to be able to predict whether the pregnancy will end in the death of the baby inside the womb (fetal death) or such serious concerns about the baby's wellbeing that it would need to be delivered extremely prematurely (before 28 weeks of gestation i.e. 3 months prematurely)?
	Which is more important: correctly identifying women who WILL have this outcome (true positives, sensitivity) or correctly identifying women who will NOT have this outcome (true negatives, specificity)?
	How important is it to be able to predict whether the blood flow measurements in the umbilical cord (umbilical artery Dopplers) will become abnormal if they were normal at first?<br><br>Note: The umbilical arteries take blood from the baby back to the placenta. We use Doppler ultrasound to measure the something called the 'pulsatility index' in the umbilical arteries. When this is higher than normal it suggests there is a problem with how the placenta has developed and/or how the placenta is working. If umbilical artery Dopplers are normal the baby may just be 'constitutionally' small but if the umbilical artery Dopplers are abnormal it is more likely that the baby is small because of a problem with the placenta.
	Which is more important: correctly identifying women who WILL have this outcome (true positives, sensitivity) or correctly identifying women who will NOT have this outcome (true negatives, specificity)?
	How important is it to be able to predict whether the pregnancy will end in the live birth of the baby at term (37 weeks of pregnancy or more)?
	Which is more important: correctly identifying women who WILL have this outcome (true positives, sensitivity) or correctly identifying women who will NOT have this outcome (true negatives, specificity)?

References

HADLOCK, F. P., HARRIST, R. B., SHARMAN, R. S., DETER, R. L. & PARK, S. K. 1985. Estimation of fetal weight with the use of head, body, and femur measurements--a prospective study. *Am J Obstet Gynecol,* 151**,** 333-7.

KHONG, T. Y., MOONEY, E. E., ARIEL, I., BALMUS, N. C., BOYD, T. K., BRUNDLER, M. A., DERRICOTT, H., EVANS, M. J., FAYE-PETERSEN, O. M., GILLAN, J. E., HEAZELL, A. E., HELLER, D. S., JACQUES, S. M., KEATING, S., KELEHAN, P., MAES, A., MCKAY, E. M., MORGAN, T. K., NIKKELS, P. G., PARKS, W. T., REDLINE, R. W., SCHEIMBERG, I., SCHOOTS, M. H., SEBIRE, N. J., TIMMER, A., TUROWSKI, G., VAN DER VOORN, J. P., VAN LIJNSCHOTEN, I. & GORDIJN, S. J. 2016. Sampling and Definitions of Placental Lesions: Amsterdam Placental Workshop Group Consensus Statement. *Arch Pathol Lab Med,* 140**,** 698-713.

MARSAL, K. 2009. Obstetric management of intrauterine growth restriction. *Best Practice & Research Clinical Obstetrics and Gynaecology,* 23**,** 857-870.

MARSAL, K., PERSSON, P. H., LARSEN, T., LILJA, H., SELBING, A. & SULTAN, B. 1996. Intrauterine growth curves based on ultrasonically estimated foetal weights. *Acta Paediatr,* 85**,** 843-8.

SCHAFFER, A. C. & STAUDACH, A. 1997. *RE: Doppler-Referenzkurven.*

STIRNEMANN, J., VILLAR, J., SALOMON, L. J., OHUMA, E., RUYAN, P., ALTMAN, D. G., NOSTEN, F., CRAIK, R., MUNIM, S., CHEIKH ISMAIL, L., BARROS, F. C., LAMBERT, A., NORRIS, S., CARVALHO, M., JAFFER, Y. A., NOBLE, J. A., BERTINO, E., GRAVETT, M. G., PURWAR, M., VICTORA, C. G., UAUY, R., BHUTTA, Z., KENNEDY, S. & PAPAGEORGHIOU, A. T. 2017. International estimated fetal weight standards of the INTERGROWTH-21st Project. *Ultrasound Obstet Gynecol,* 49**,** 478-486.
